# Inhibiting BCKDK in triple negative breast cancer suppresses protein translation, impairs mitochondrial function, and potentiates doxorubicin cytotoxicity

**DOI:** 10.1038/s41420-021-00602-0

**Published:** 2021-09-15

**Authors:** Dipsikha Biswas, Logan Slade, Luke Duffley, Neil Mueller, Khoi Thien Dao, Angella Mercer, Shanmugasundaram Pakkiriswami, Yassine El Hiani, Petra C. Kienesberger, Thomas Pulinilkunnil

**Affiliations:** 1 Department of Biochemistry and Molecular Biology, Faculty of Medicine, Dalhousie University, Dalhousie Medicine New Brunswick, Saint John, New Brunswick Canada; 2 Department of Physiology and Biophysics, Faculty of Medicine, Dalhousie University, Halifax, Nova Scotia Canada

**Keywords:** Cancer metabolism, Nutrient signalling

## Abstract

Triple-negative breast cancers (TNBCs) are characterized by poor survival, prognosis, and gradual resistance to cytotoxic chemotherapeutics, like doxorubicin (DOX). The clinical utility of DOX is limited by its cardiotoxic and chemoresistant effects that manifest over time. To induce chemoresistance, TNBC rewires oncogenic gene expression and cell signaling pathways. Recent studies have demonstrated that reprogramming of branched-chain amino acids (BCAAs) metabolism facilitates tumor growth and survival. Branched-chain ketoacid dehydrogenase kinase (BCKDK), a regulatory kinase of the rate-limiting enzyme of the BCAA catabolic pathway, is reported to activate RAS/RAF/MEK/ERK signaling to promote tumor cell proliferation. However, it remains unexplored if BCKDK action remodels TNBC proliferation and survival per se and influences susceptibility to DOX-induced genotoxic stress. TNBC cells treated with DOX exhibited reduced BCKDK expression and intracellular BCKAs. Genetic and pharmacological inhibition of BCKDK in TNBC cell lines also showed a similar reduction in intracellular and secreted BCKAs. BCKDK silencing in TNBC cells downregulated mitochondrial metabolism genes, reduced electron complex protein expression, oxygen consumption, and ATP production. Transcriptome analysis of BCKDK silenced cells confirmed dysregulation of mitochondrial metabolic networks and upregulation of the apoptotic signaling pathway. Furthermore, BCKDK inhibition with concurrent DOX treatment exacerbated apoptosis, caspase activity, and loss of TNBC proliferation. Inhibition of BCKDK in TNBC also upregulated sestrin 2 and concurrently decreased mTORC1 signaling and protein synthesis. Overall, loss of BCKDK action in TNBC remodels BCAA flux, reduces protein translation triggering cell death, ATP insufficiency, and susceptibility to genotoxic stress.

## Abstract (web summary)

Proposed mechanism. **A** Doxorubicin (DOX) targets the BCAA catabolic pathway in TNBCs, by downregulating BCKDK and augmenting clearance of intracellular BCKAs. **B** Genetic or pharmacological (high BT2 concentration) inhibition of BCKDK results in increased cell death, decreased intracellular BCKAs, dysregulated mitochondrial function, ATP insufficiency, SESN2 activation, and inhibition of mTORC1 signaling and protein synthesis. C BCKDK inhibition (siRNA mediated or low-BT2 concentration) exacerbates DOX-induced cytotoxicity and caspase activity.

## Graphical abstract

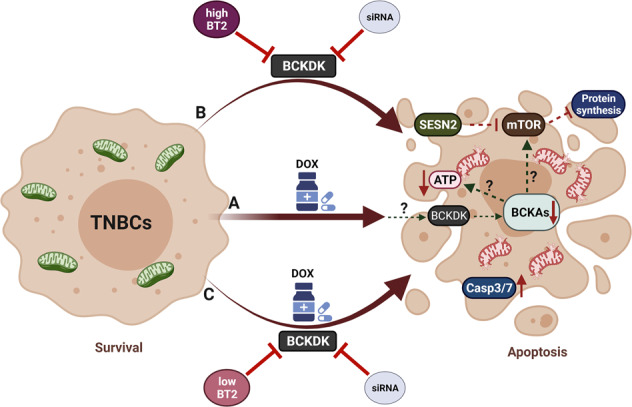

## Introduction

Triple-negative breast cancers (TNBCs) are an aggressive subtype of breast cancer constituting 10–15% of all breast cancers and are defined by the lack of expression of estrogen receptor (ER), progesterone receptor (PR), and the human epidermal growth factor receptor 2 (HER2) [1]. Depending on the severity and stage of the disease [2], TNBCs are generally treated in different combinations with genotoxic chemotherapies, including anthracyclines, taxanes, docetaxel, and cyclophosphamides. Doxorubicin (DOX) is an anthracycline class of cytotoxic chemotherapeutic that induces TNBC remission in about 30% of patients [3]; however, prolonged treatment with higher doses of DOX results in cardiotoxicity [4], limiting its efficacy. Breast cancer cells can also reprogram cellular metabolism and function to evade toxicity of chemotherapeutic agents thereby exhibiting chemoresistance [5]. Metabolic maladaptation in TNBCs includes increased reliance on the pentose phosphate pathway for NADPH, lysosomal and mitochondrial drug sequestration, altered nutrient metabolism, mitochondrial reprogramming, increased polyamine biosynthesis, glycolysis and glutaminolysis, and amino acid uptake for protein biosynthesis [5]. Therefore, identifying mechanisms by which TNBCs survive DOX cytotoxicity and targeting the vulnerabilities in metabolic pathways that render TNBCs resistant to chemotherapy will enable development of concurrent therapies co-administered with lower doses of DOX.

Amino acid uptake and utilization facilitate the uninterrupted synthesis of TCA intermediates and proteins for increased nitrogen and proliferative demand of TNBCs [6]. Cancer cells tightly control the regulation of branched-chain amino acids (BCAAs), namely leucine, isoleucine, and valine, which contribute to 20% of the TCA cycle intermediates. Besides direct incorporation into proteins, catabolism of BCAAs produces intermediates, like glutamate, that are vital for driving TNBC growth and survival [7]. BCAAs are reversibly transaminated to branched-chain ketoacids (BCKAs) by branched-chain aminotransferase (BCAT). BCKAs are further catabolized by branched-chain alpha-ketoacid dehydrogenase (BCKDH), ultimately generating TCA cycle intermediates. BCKDH is inhibited by phosphorylation at Ser 293 residue by the kinase, branched-chain alpha-ketoacid dehydrogenase kinase (BCKDK), while Mg^2+^/Mn^2+^ Dependent 1K (PPM1K) protein phosphatase [7] induces dephosphorylation. Recently, in glioblastomas and pancreatic cancer, a role for BCKAs was uncovered in metabolic homeostasis [8], immune suppression, and cell death evasion [9]. Although the role of BCKDH complex and BCAT isoforms (cytosolic BCAT1 or mitochondrial BCAT2) have been widely explored in different cancers [10, 11], the role of BCKDK remains mostly elusive. Recent studies have identified novel phosphorylation sites on BCKDK that stabilize BCKDK activity and influence tumor cell proliferation and survival [12, 13]. The reprogramming of BCAA metabolism produces intermediates that rewire oncogenic gene expression and cell signaling pathways in TNBC [11]. However, the importance of BCKDK in regulating the BCAA pool and their flux into BCKAs as well as its impact on TNBC growth and survival merits investigation.

In the current study, we investigated the role of BCKDK in TNBC survival and metabolism and examined whether DOX’s anticancer effect involves targeting the BCAA catabolic pathway. DOX treatment at 2 µM suppressed BCKDK, accelerated intracellular BCKAs clearance and significantly increased mRNA and protein levels of BCAA degradation enzymes in two TNBC cell lines. Transcriptome analysis of MDA-MB231 cells with BCKDK silencing revealed an increment in apoptotic signaling and deregulated mitochondrial function. Genetic and pharmacological inhibition of BCKDK sensitized TNBCs to DOX-induced cytotoxicity. BCKDK inhibition suppressed mitochondrial function, reduced nascent protein synthesis, and increased sestrin 2 (SESN2) expression, a negative regulator of protein synthesis and cell proliferation. Collectively, our data highlight the role of DOX in regulating BCAA catabolism and identifies BCKDK as a novel DOX-sensitive target in TNBCs. Targeting BCKDK can be an attractive therapeutic target in regulating TNBC survival, proliferation, and potentiating chemosensitivity to DOX.

## Results

### Altered BCAA catabolic enzyme expression in TNBCs is sensitive to DOX treatment

**Fig. 1:**
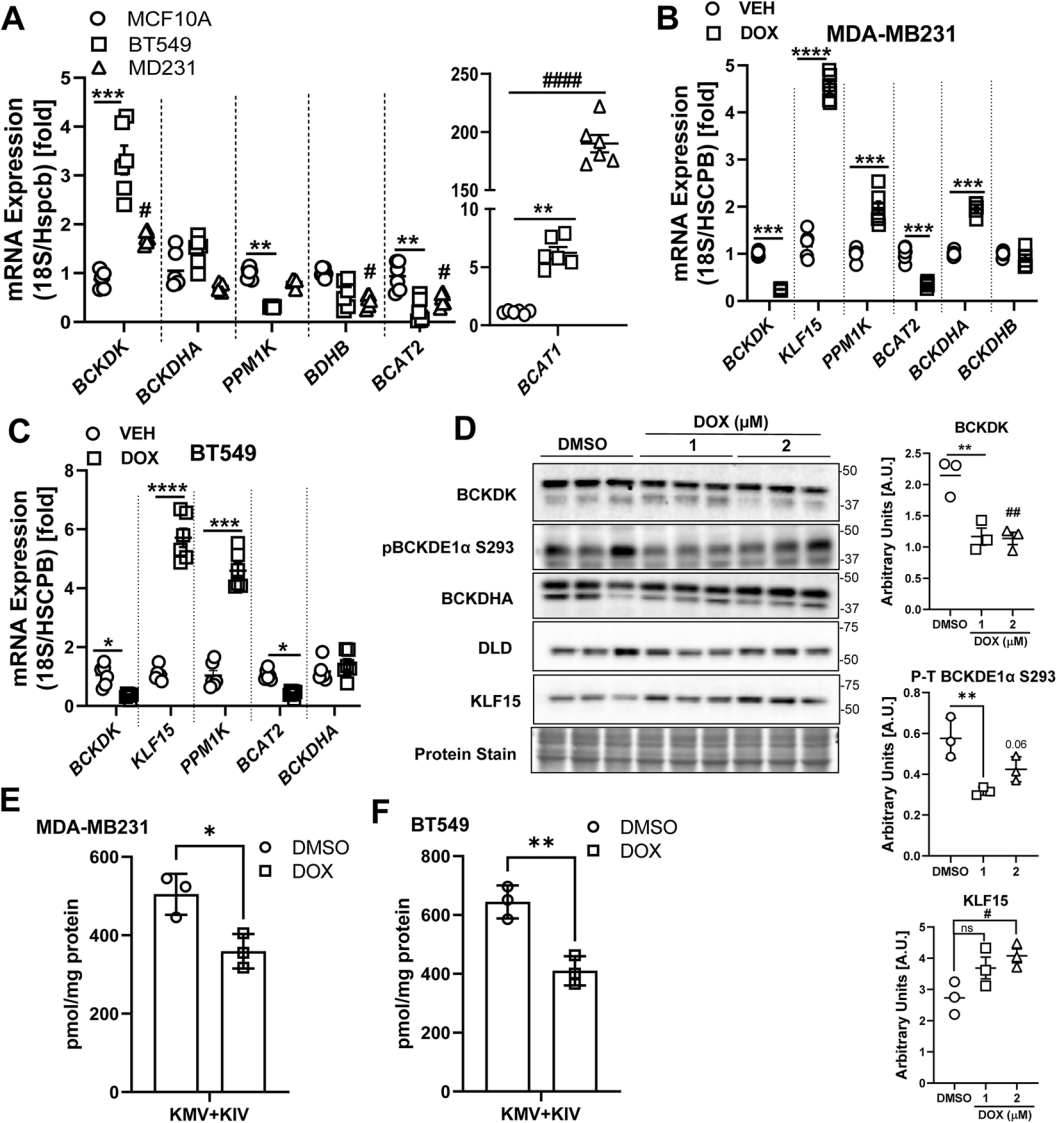
DOX suppresses BCKDK expression and augments BCAA oxidation enzyme expression in TNBCs. **A** Quantification of *BCKDK*, *BCKDHA*, *PPM1K*, *BCKDHB*, *BCAT2*, and *BCAT1* mRNA expression corrected to 18S/HSPCB reference genes in MCF10A, BT549, and MDA-MB231 cells. **B**, **C** BCAA catabolic enzyme expression in TNBCs treated with 2 µM DOX for 18 h. Quantification of *BCKDK*, *BCKDHA*, *PPM1K*, *BCKDHB*, *BCAT2*, and *KLF15* mRNA expression corrected to 18S/HSPCB reference genes in MDA-MB231 (**B**) and BT549 (**C**) cells. **D** Immunoblot and densitometric analysis of BCKDK, total and phosphorylated BCKDHA E1α Ser 293, DLD and KLF15 in MDA-MB231 cells treated with 1 and 2 µM DOX for 18 h. Quantifications are from three independent experiments. Statistical analysis was performed using a two-way ANOVA followed by a Tukey’s multiple comparison test; *p < 0.05, **p < 0.01, ****p < 0.0001 as indicated. *MCF10A vs. BT549 or DMSO vs. 1 µM DOX, #MCF10A vs. MDA-MB231 or DMSO vs. 2 µM DOX. **E**, **F** UPLC MS/MS analysis of intracellular BCKAs in **E** MDA-MB231 and **F** BT549 cells. All data are presented as mean ± S.D. Statistical analysis was performed using a Student’s *t* test; *p < 0.05, **p < 0.01, ****p < 0.0001 as indicated.

BCAA catabolic enzyme expression was examined in two TNBC cell lines, BT549 and MDA-MB231. In agreement with prior studies [14], we found a striking upregulation of *BCAT1* mRNA levels, specifically in MDA-MB231 cells, while *BCAT2* mRNA (Fig. 1A) and protein (Fig. S1B) levels were significantly downregulated in both BT549 and MDA-MB231 cells when compared with normal breast epithelial cell line, MCF10A. Treatment with 2 µM DOX reduced *BCAT1* mRNA expression in BT549 cells (Fig. S1D) and further reduced *BCAT2* mRNA expression in both TNBCs (Fig. 1B, C). BCKDHA and BCKDHB protein levels were significantly downregulated in TNBC cell lines (Fig S1B), although their mRNA levels were selectively reduced in the MDA-MB231 cells (Fig. 1A). DOX treatment increased *BCKDHA* mRNA levels in MDA-MB231 cells (Fig. 1B). In the TNBC cells, mRNA (Fig. S1A) and protein (Fig. S1B) expression of *KLF15*, the transcriptional activator of the BCAA pathway, was suppressed. DOX treatment significantly increased *KLF15* mRNA (Fig. 1B-C) expression in both TNBCs and KLF15 protein content specifically (Fig. 1D) in MDA-MB231 cells. The BCKDH phosphatase, *PPM1K*, mRNA levels were also decreased in TNBCs (Fig. 1A) and were upregulated in response to DOX (Fig. 1B, C). mRNA expression of distal enzymes, *ACADSB*, *HADHA*, and *HIBCH* were unchanged (Fig. S1A), however, DOX increased the mRNA expression of *HADHA* in both TNBCs (Fig. S1C, D). These data suggest that in TNBCs, expression of proximal BCAA catabolic enzymes are suppressed, and treatment with DOX counteracts this suppression, likely affecting BCAA catabolism. Since the role of BCAT and BCKDH were examined in prior studies, we focussed on studying the role of BCKDK. This kinase regulates the flux of BCKAs towards oxidation by inactiving phosphorylation of BCKDH. *BCKDK* transcripts were markedly upregulated in BT549 and moderately increased in MDA-MB231 cells (Fig. 1A) compared to noncancerous MCF10A cells. Although BCKDK protein expression was augmented in BT549, it remained comparable (*p* = 0.08) to MCF10A cells and MDA-MB231 cells (Fig. S1B). The ratio of phosphorylated to total BCKDE1α S293 content was increased in BT549 but not in MDA-MB231 and MCF10A cells (Fig. S1B). DOX treatment reduced *BCKDK* mRNA levels in both TNBCs (Fig. 1B, C). 2 µM DOX treatment reduced BCKDK protein levels and the corresponding phosphorylated BCKDE1α S293 levels in MDA-MB231 (Fig. 1D) however, in BT549 cells, DOX only decreased the latter (Fig. S1E). Furthermore, DOX treatment significantly reduced the intracellular BCKAs (KIV and KMV) levels in both TNBC cell lines (Fig. 1E, F). Since BCKDK expression was altered in TNBCs and regulated by DOX, we next examined the impact of altered BCKDK on TNBC metabolism and proliferation.

### Downregulating BCKDK inhibits proliferation, promotes apoptosis, and potentiates DOX-mediated cell death

**Fig. 2:**
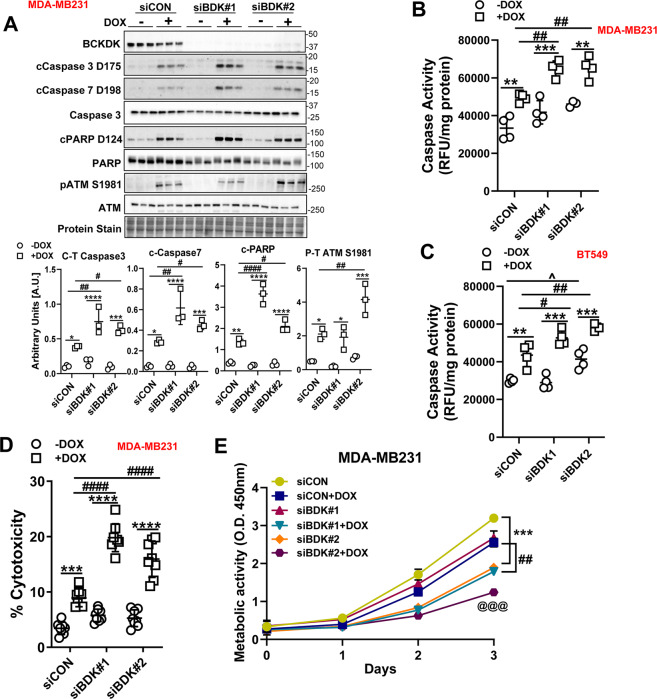
Silencing BCKDK exacerbates DOX-mediated cell death in TNBCs. **A** Immunoblot and densitometric analysis of BCKDK, total and cleaved Caspase 3, cleaved Caspase 7, total and cleaved PARP, total and phosphorylated ATM Ser 1981 in MDA-MB231 cells transfected with siCON, siBDK#1, or siBDK#2 for 72 h followed by 2 µM DOX or DMSO for 18 h. **B**, **C** MDA-MB231 (**B**) and BT549 (**C**) cells were transfected with siCON, siBDK#1, or siBDK#2 for 72 h followed by 2 µM DOX or DMSO treatment for 18 h and analyzed for Caspase 3 activity. **D**, **E** MDA-MB231 cells transfected with siCON, siBDK#1, or siBDK#2 for 72 h followed by 2 µM DOX or DMSO treatment for 18 h and analyzed for **D** LDH release in the media, and **E** metabolic activity measured by CCK8 assay. Quantifications are from three independent experiments. **A**–**D**, *within groups (DMSO vs DOX), #between DOX groups (siCON vs siBDK#1 or vs siBDK#2), **E**, *siCON vs siBDK#2, #siCON+DOX vs siBDK#1+DOX, @siCON+DOX vs siBDK#2+DOX. Data presented as mean ± S.D. Statistical analysis was performed using a two-way ANOVA followed by a Tukey’s multiple comparison test; *p < 0.05, **p < 0.01, ****p < 0.0001 as indicated.

To decipher whether BCKDK silencing alters metabolism and survival of TNBCs, MDA-MB231 cells were treated with either control siRNA (siCON) or siRNAs targeting BCKDK exon 2 (siBDK#1) or exon 7 (siBDK#2). BCKDK silencing was confirmed by decreased mRNA (Fig. S2A) and protein (Fig. S2B) levels. Silencing BCKDK using siBDK#2 resulted in reduced cell count at 24 and 48 h following transfection, while siBDK#1 showed reduced cell count at 48 h (Fig. S2C), confirming that TNBC requires functional BCKDK for survival and proliferation. BCKDK deletion also resulted in significant upregulation of cleaved caspase 3 content (Fig. S2B), indicating increased cell death. BCKDK knockdown exacerbated DOX mediated increases in cleaved caspase 3, caspase 7, and PARP levels in MDA-MB231 cells (Fig. 2A). Moreover, in BCKDK depleted cells, exposure to DOX increased phosphorylation of the DNA damage marker, ataxia-telangiectasia mutated (ATM), suggesting increased genomic instability and apoptosis (Fig. 2A). Similar increases were observed in cleaved caspase 3/7 and Bax expression in DOX-treated BT549 cells with BCKDK silencing (Fig. S2D). To rule out off-target effects of siRNA-mediated gene silencing, we also performed the experiments with adenoviral-mediated knockdown of BCKDK in both MDA-MB231 and BT549 cells and observed similar increases in cleaved caspase levels in response to DOX (Fig. S3A). Changes in protein levels were also reflected in increased caspase activity in BCKDK depleted MDA-MB231 (Fig. 2B) and BT549 (Fig. 2C) cells treated with DOX. Cytotoxicity of DOX was evident by increased lactate dehydrogenase (LDH) release into the media, which was exacerbated further following BCKDK silencing (Fig. 2D). Antibody array assay of apoptosis-associated proteins showed decreased levels of the antiapoptotic protein, survivin, and increased content of pro-apoptotic protein, BIM, and cytochrome C in BCKDK silenced MDA-MB231 cells. Also, in MDA-MB231 cells, DOX-induced increases in cytochrome C, Fas, BIM, and caspase 8 expression were potentiated (Fig. S3B).

We next examined whether the increased cytotoxic response to DOX following BCKDK knockdown was associated with decreased cell viability. Resazurin-based cell viability was performed in MDA-MB231 cells transduced with shBCKDK and treated with DOX for 18 h followed by a chase in drug-free media for 48 h. Knockdown of BCKDK resulted in a significant decrease in cell viability that further declined upon exposure to DOX (Fig. S3C). In addition, a CCK8 assay was performed to determine the metabolic activity of the BCKDK silenced MDA-MB231 cells in response to DOX. BCKDK knockdown alone reduced cell proliferation by 2 d (siBDK#2) or 3 d (siBDK#1), which was potentiated further by DOX treatment (Fig. 2E).

**Fig. 3:**
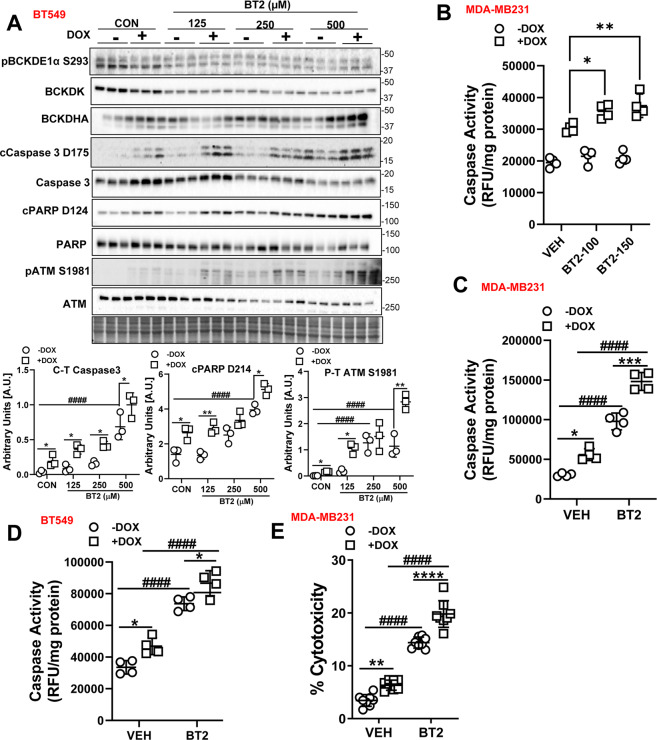
BT2-mediated BCKDK inhibition induces cell death and potentiates DOX-mediated apoptosis in TNBCs. **A** Immunoblot and densitometric analysis of total and phosphorylated BCKDE1α Ser 293, BCKDK, total and cleaved Caspase 3, total and cleaved PARP, total and phosphorylated ATM Ser 1981 in BT549 cells pre-treated with 125, 250, and 500 µM BT2 for 20 h followed by 2 µM DOX or DMSO treatment for 18 h. **B** Measurement of Caspase 3 activity in BT549 cells pre-treated with 100 µM and 150 µM BT2 for 20 h followed by 2 µM DOX or DMSO treatment for 18 h. Caspase 3 activity was measured in MDA-MB231 (**C**) and BT549 (**D**) cells and LDH release into the media was measured in MDA-MB231 cells (**E**) pre-treated with 500 µM BT2 for 20 h followed by 2 µM DOX or DMSO treatment for 18 h. Quantifications are from three independent experiments. **A**, *within groups (DMSO vs DOX), #between DOX groups (siCON vs siBDK#1 or vs siBDK#2); **C**–**E**, *within groups (DMSO vs DOX), #between groups (VEH vs BT2 and VEH+DOX vs BT2+DOX). Data presented as mean ± S.D. Statistical analysis was performed using a two-way ANOVA followed by a Tukey’s multiple comparison test; *p < 0.05, **p < 0.01, ****p < 0.0001 as indicated.

We next recapitulated the cytotoxic effects of BCKDK silencing by treating cells with the BCKDK inhibitor BT2 in the presence and absence of DOX. At lower concentrations, BT2 modestly potentiated DOX’s induced increases in cleaved caspase-3 and cleaved PARP levels with the maximal effects observed at 500 µM. Regardless of the concentration used, BT2 also potentiated DOX’s effect on phosphorylating DNA damage marker, ATM in BT549 cells (Fig. 3A). Although the caspase 3 protein levels were modestly affected at lower concentrations, BT2 exacerbated DOX mediated increases in caspase activity at 100 and 150 µM (Fig. 3B). At a concentration of 500 µM, BT2 treatment alone resulted in increased levels of cleaved PARP, caspase 3, and phosphorylated ATM in both BT549 (Fig. 3A) and MDA-MB231 cells (Fig S4A), as well as cleaved caspase 7 in MDA-MB231 cells (Fig. S4A). Concomitant with the protein expression, BT2 treatment per se markedly increased caspase activity and exacerbated caspase activity in both DOX treated MDA-MB231 (Fig. 3C) and BT549 (Fig. 3D) cells. Moreover, in MDA-MB231 cells BT2 alone increased LDH release, and this effect was further exacerbated in the presence of DOX (Fig. 3E).

### Silencing BCKDK alters TNBC transcriptome

**Fig. 4:**
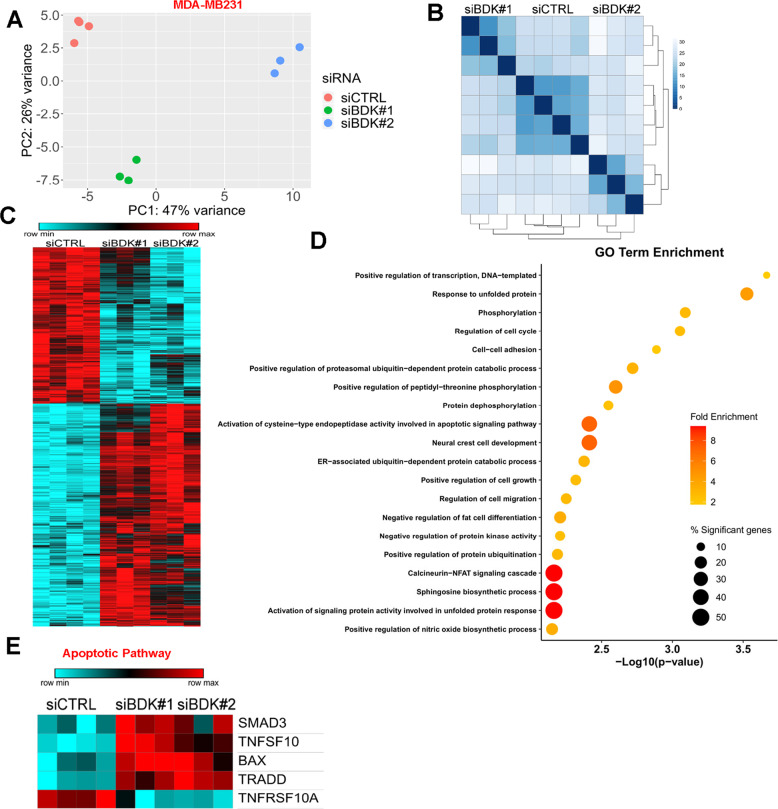
Differential enrichment of genes related to metabolism and cell death pathways in MDA-MB-231 cells with BCKDK knockdown. **A** Principle component analysis plot for all gene expressions from MDA-MB231 cells treated with both siRNAs targeting BCKDK. **B** Distance matrix clustering for all gene expressions from MDA-MB231 cells. **C** Heatmap for differentially expressed genes from MDA-MB231 cells. **D** Top 20 most significantly enriched gene ontology (GO) terms from BCKDK knockdown induced differentially expressed genes. Color represents the fold enrichment statistic, and size represents the percentage of the differentially expressed genes in the gene set compared with the total gene set size. **E** Heatmap for genes involved in apoptosis which were significantly differentially regulated by BCKDK knockdown in MDA-MB231 cells.

We next aimed to identify metabolic and signaling pathway networks regulated by BCKDK to influence TNBC cell death and proliferation. MDA-MB231 cells depleted of BCKDK were subjected to RNA-Seq transcriptomic analysis. Principle component analysis and distance clustering showed that the transcriptome of cells treated with siBDK#1 and siBDK#2 displayed little similarity (Fig. 4A, B), thus we classified genes as differentially expressed compared with control if the adjusted *P*-value was below 0.01 for each siRNA individually and the fold change was occurring in the same direction. This method identified 1024 genes, which were differentially expressed by both BCKDK siRNAs and over-represented Gene Ontology (GO) were determined using DAVID (Fig. 4C). Among the top 20 GO terms identified, activation of response to unfolded protein response and positive regulation of ubiquitin-dependent protein catabolism appeared multiple times (Fig. 4D). These processes play a crucial role in regulating apoptosis by direct or indirect targeting of important regulators of apoptosis and caspases [15, 16]. Indeed, the GO term related to activation of caspases, the cysteine-type endopeptidase, was enriched in the differentially expressed genes from BCKDK knockdown cells (Fig. 4D), consistent with our data (Figs. 2 and 3). Genes annotated to this GO term include the initiator or activator caspases, including CASP2, CASP8, CASP9, and CASP10, also called initiator (or apical, or activator) caspases, suggesting that BCKDK silencing is plausibly sensitizing TNBC to cell death. Indeed, differentially expressed genes involved in death receptor signaling were upregulated after BCKDK knockdown, including TNF-related apoptosis-inducing ligand (TRAIL/TNFSF10) and TNFRSF1A associated via death domain (TRADD) (Fig. 4E). The expression of pro-apoptotic factors, Bax, and Smad3, involved in activating TGF-β-induced apoptosis, increased in BCKDK depleted cells (Fig. 4E), indicating augmented pro-apoptotic gene expression.

### BCKDK inhibition decreases intracellular and secreted BCKAs

We next ascertained whether BCKDK silencing altered mRNA expression of BCAA catabolic enzymes, which facilitate BCKA oxidation. RNA-Seq analysis revealed that BCKDK knockdown resulted in increased expression of genes involved in BCKA oxidation, specifically *BCKDHA*, methylmalonyl-CoA epimerase (*MCEE*), propionyl-CoA carboxylase (*PCCB*), *HADHB* and *ACADM* (Fig. S5A). Loss of BCKDK led to reduced expression of *BCAT1* and *HIBCH* (Fig. S5A), candidate genes augmented in several cancers [17]. Quantitative polymerase chain reaction (qPCR) analysis revealed significant increases in the BCKDHA and KLF15 mRNA expression in MDA-MB231 (Fig. S5B), although it was only observed with siBDK#1. No changes were observed in the mRNA expression of BCAA catabolic enzymes in the BT549 cells (Fig. S5C). Contrary to BCKDK silencing, BT2 treatment resulted in significant increases in *KLF15*, *BCKDHA*, *BCKDHB*, *HIBCH*, and *HIBADH* mRNA levels in BT549 (Fig. S5D). Next we determined if increased expression of BCAA catabolizing genes resulted in reduced intracellular accumulation and secretion of BCKAs. Adenoviral knockdown of BCKDK in MDA-MB231 cells resulted in decreased intracellular BCKAs accumulation as revealed by ultraperformance liquid chromatography-tandem mass spectrometry analysis (Fig. S5E). A significant reduction in total BCKAs was observed in cell lysates and media from BT2 treated MDA-MB231 cells (Fig. S5F, G). Previous studies have demonstrated the role of tumor secreted BCKAs in the proliferation and immune suppression of cancer cells [8, 9]. Our data suggest that the mechanism by which BCKDK depletion arrests TNBC proliferation likely involves increased BCKA oxidation with a concomitant reduction in accumulation and secretion of BCKAs.

### BCKDK inhibition decreased ETC complex protein expression and impaired mitochondrial function

**Fig. 5:**
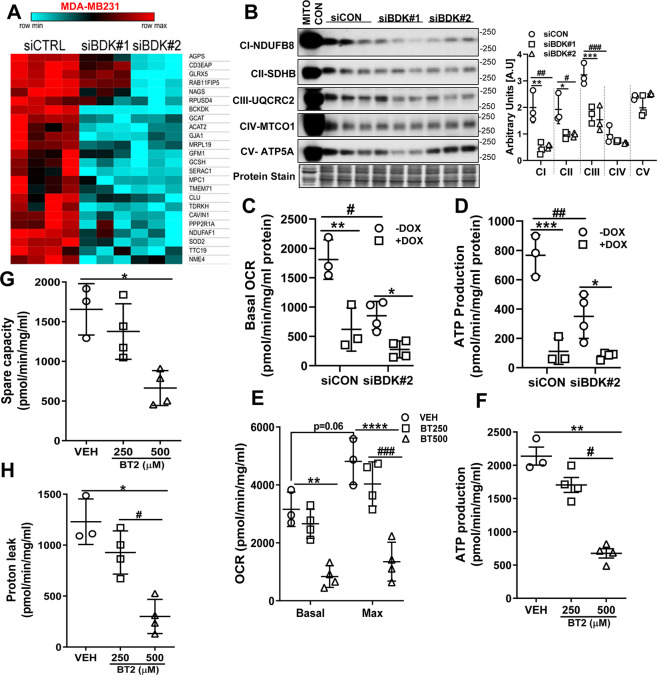
BCKDK inactivation suppresses mitochondrial function and complex protein expression. **A** Heatmap for genes involved in mitochondrial biogenesis, structure, and function significantly differentially regulated by BCKDK knockdown. **B** Immunoblot and densitometric quantification of complex I–V proteins in MDA-MB231 whole cell lysates transfected with siCON, siBDK#1, or siBDK#2 for 72 h. Data presented as mean ± S.D. Statistical analysis was performed using a two-way ANOVA followed by a Tukey’s multiple comparison test; *p < 0.05, **p < 0.01, ****p < 0.0001 as indicated. **C**–**H** Mitochondrial function measured in BCKDK depleted MDA-MB231 cells using extracellular flux analyzer in the presence of 25 mM glucose. **C** Basal OCR, **D** ATP production measured in MDA-MB231 cells transfected with siCON or siBDK#2. **E** Basal and maximal OCR, **F** ATP production, **G** spare capacity, and **H** proton leak measured in MDA-MB231 cells treated with 250 or 500 µM BT2 for 20 h. **B**, *siCON vs siBDK#1, #siCON vs siBDK#2; **C**–**D**, *within groups (DMSO vs DOX), #between groups (siCON vs siBDK#2); **E**–**H**, *VEH vs 250μM BT2, #VEH vs 500μM BT2. Data presented as mean ± S.D. Statistical analysis was performed using a was performed using Student’s *t* test; *p < 0.05, **p < 0.01, ****p < 0.0001 as indicated.

**Fig. 6:**
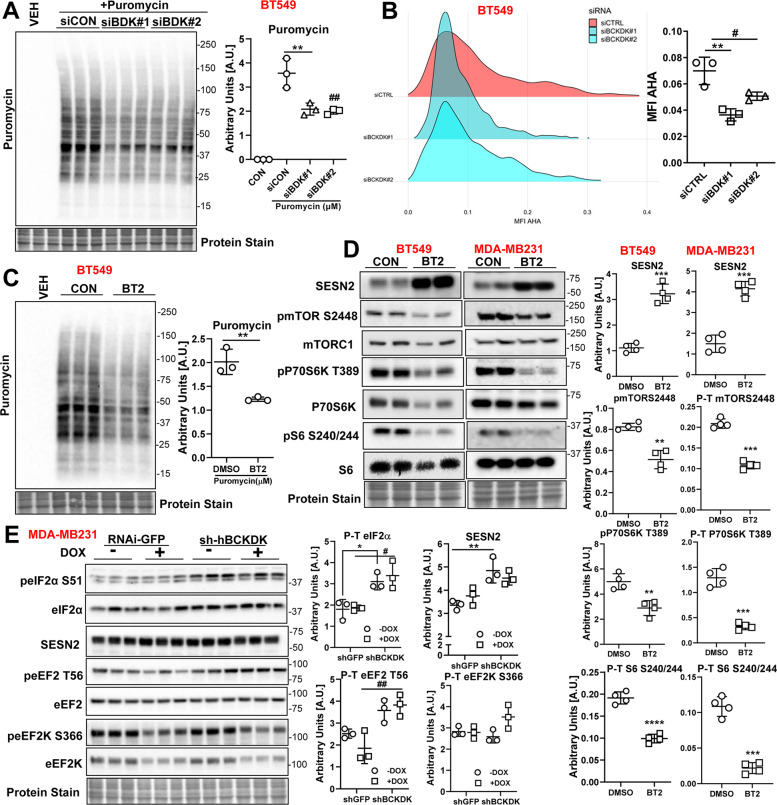
BCKDK inhibition decreases protein synthesis and mTOR signaling in TNBCs. **A** BT549 was transfected with siCON, siBDK#1, or siBDK#2 for 72 h followed by incubation with 1 μM puromycin for 30 min. Immunoblot and densitometric analysis of puromycin incorporation. **B** Fluorogram and quantification of AHA incorporation in BT549 cells transfected with siCON, siBDK#1, or siBDK#2. C Immunoblot and densitometric analysis of puromycin incorporation in BT549 cells treated with 500 µM BT2 for 20 h followed by incubation with 1 μM puromycin for 30 min. The graph represents mean ± S.D., *n* = 3, *p < 0.05 was performed using Student’s *t* test. **D** Immunoblot and densitometric analysis of SESN2, total and phosphorylated mTOR S2448, total and phosphorylated P70S6K T389, and total to phosphorylated S6 S240/244 in BT549 and MDA-MB231 cells treated with 500 µM BT2 for 20 h. Data presented as mean ± S.D. Statistical analysis was performed using Student’s *t* test; *p < 0.05, **p < 0.01, ****p < 0.0001 as indicated. Immunoblot and densitometric analysis of total and phosphorylated eIF2α S51, Sesn2, total and phosphorylated eEF2 T56, total and phosphorylated eEF2K S266, total and phosphorylated AKT S473, and AKT T308 in MDA-MB231 cells transduced with shGFP or shBCKDK for 48 h followed by 2 µM DOX or DMSO treatment for 18 h. Quantifications are from three independent experiments. **A**–**B**, *siCON vs siBDK#1, #siCON vs siBDK#2; **E**, *shGFP vs sh-hBCKDK, #shGFP+DOX vs sh-hBCKDK+DOX. Data presented as mean ± S.D. Statistical analysis was performed using a two-way ANOVA followed by a Tukey’s multiple comparison test; *p < 0.05, **p < 0.01, ****p < 0.0001 as indicated.

To explain the increased clearance of BCKAs in TNBCs treated with DOX in conjunction with BCKDK silencing, we next examined mitochondrial function. Permeabilized and damaged mitochondria generate reactive oxygen species in a complex I and II-dependent manner [18] and when recognized by activated caspase is targeted for destruction. Transcriptomic analysis of BCKDK depleted MDA-MB231 cells revealed a marked downregulation of genes involved in mitochondrial function and biogenesis (Fig. 5A). For instance, BCKDK silencing reduced nucleoside diphosphate kinase (NME4) and clusterin (Clu) gene expression, which are involved in promoting redistribution of cardiolipin to prevent mitochondrial permeabilization and suppressing Bax dependent release of cytochrome C, respectively (Fig. 5A). Genes involved in mitochondrial translation, mitochondrial ribosome construction, and abundances of 16S mt-rRNA, such as G elongation factor mitochondrial 1 (GFMI), mitochondrial ribosomal protein L19 (MRPL19) and RNA pseudouridine synthase D4 (RPUSD4), were also downregulated upon BCKDK silencing (Fig. 5A). Furthermore, genes involved in complex I assembly (NADH:ubiquinone oxidoreductase assembly factor 1, NDUFAF1), the formation of complex III (tetratricopeptide repeat domain 19, TTC19), suppression of ROS (superoxide dismutase 2, SOD2), and synthesis of nucleotide triphosphates and mitochondrial respiration (NME4, serine active site containing 1, SERAC) were downregulated in BCKDK depleted cells (Fig. 6A). In addition, mitochondrial genes involved in pyruvate transport (mitochondrial pyruvate carrier 1, MPC1), glycine metabolism (glycine cleavage system protein H, GCSH, and glycine c-acetyltransferase, GCAT) and ether lipid synthesis and intracellular cholesterol trafficking (peroxisomal alkylglycerone phosphate synthase, AGPS, and mitochondrial SERAC1, respectively) were also downregulated upon BCKDK silencing (Fig. 5A). Consistent with the transcriptomic data, silencing BCKDK in MDA-MB231 cells decreased complex I, complex II, and complex III protein levels (Fig. 5B). Extracellular flux analysis studies revealed reduced basal oxygen consumption rates (OCR) (Fig. 5C), ATP production (Fig. 5D), and non-mitochondrial respiration (Fig. S6A) in the BCKDK silenced cells. Inhibiting BCKDK by BT2 also resulted in reduced basal and maximal OCR (Fig. 5E) and ATP production (Fig. 5F), indicating impaired mitochondrial oxidative metabolism. Lower OCR reduced spare respiratory capacity (Fig. 5G), proton leak (Fig. 5H) and low non-mitochondrial respiration (Fig S6B), suggestive of impaired adaptation to metabolic changes. Mitochondrial DOX accumulation can also disrupt mitochondrial architecture and oxidative capacity [19]. We determined whether BCKDK silencing in MDA-MB231 cells can potentiate DOX’s effects, exacerbating mitochondrial dysfunction. DOX treatment reduced basal OCR (Fig. 5C), ATP production (Fig. 5D), and non-mitochondrial respiration (Fig. S6A), but it was not significantly potentiated upon BCKDK silencing. Unlike TNBCs, normal breast epithelial cells, MCF10A, when treated with BT2, did not show suppressed mitochondrial respiration or ATP production (Fig. S6C–G). Although increased BCKA clearance with compromised mitochondria appear counter intuitive, we believe that the accelerated BCAA catabolism results in dysfunctional mitochondria in the BCKDK deficient TNBCs.

### Silencing BCKDK reduces protein synthesis in TNBCs

Previous studies have demonstrated that induction of ER stress and mitochondrial dysfunction [20] induces apoptosis [21] and inhibits protein translation and synthesis. As measured by puromycin incorporation, protein synthesis was reduced in BCKDK depleted BT549 cells (Fig. 6A). Similarly, azidohomoalaine (AHA) labeled nascent protein levels were decreased upon BCKDK silencing (Fig. 6B). A similar reduction in puromycin incorporation was observed in BT549 cells treated with BT2 (Fig. 6C). In both BT549 and MDA-MB231 cells, BT2 treatment resulted in a robust increase in sestrin 2 (SESN2) protein levels (Fig. 6D), a negative regulator of mammalian target of rapamycin (mTOR) and protein synthesis [22]. Consistently, mTOR signaling was suppressed in BT2 treated cells as observed by reduced phosphorylation on mTOR S2448, P70S6K T389, and S6 S240/244 (Fig. 6D). Similar increases in SESN2 levels were observed in MDA-MB231 cells adenovirally transduced with shBCKDK in the presence or absence of DOX (Fig. 6E). Further, BCKDK inhibition in the presence or absence of DOX was accompanied by increased inactivating phosphorylation of eIF2α at S51 (Fig. 6E), suggesting inhibited protein translation. BCKDK inhibition also increased phosphorylation of the elongation factor eEF2 at T56 (Fig. 6E), which correlated with suppressed protein translation.

## Discussion

TNBCs are a heterogeneous breast cancer subtype with early relapse rates, poor prognosis, and limited therapeutic options [1]. Sustaining oncogenesis in TNBCs is dependent on BCAAs which contribute to protein biosynthesis, nitrogen homeostasis, epigenetic regulation, redox balance, immune response and tumor surveillance, growth and metastasis [23, 24]. Numerous types of human cancers exhibit significant reprogramming of the BCAA metabolic pathway [11]. Nevertheless, the contribution of BCAA catabolic dysregulation in TNBC pathology, cell function, and chemoresistance merit investigation. In the current study, we observed consistently suppressed expression of BCAA catabolic enzymes along with increased mRNA expression of the regulatory kinase, BCKDK, in two TNBC cell lines. Transcriptome analysis of MDA-MB231 cells with BCKDK silencing demonstrated differential enrichment of genes involved in caspase-dependent apoptosis and mitochondrial function. BCKDK inhibition also sensitized TNBCs to DOX-induced apoptosis, mitochondrial ATP insufficiency, and suppression of mTOR-dependent protein translation by SESN2. Moreover, DOX treatment suppressed BCKDK expression and reduced intracellular BCKAs in the TNBCs. For the first time, our data propose novel crosstalk between DOX and BCAA catabolism and highlights the role of BCKDK in TNBC survival, proliferation, and DOX chemosensitivity.

Elevated intratumoral BCAAs are reported in several cancers, including breast cancer [14], PDAC [25, 26], HCC [27], and NSCLC [28], while increased BCKAs secretion is observed in glioblastomas and PDACs [8, 9]. Not only BCAA metabolites but also BCAA catabolizing enzymes have been recently shown to influence cancer outcomes [11]. Indeed, upregulation of BCAT1 is reported in glioblastomas, HCC, leukemias, osteosarcomas, ovarian and endometrial cancer [11, 27, 29]. BCAT2 was upregulated in PDAC [25, 26], luminal type breast cancer [14], NSCLC [28], while it was downregulated in HCC [27]. BCKDH was upregulated in leukemia and luminal type breast cancer [14, 30] and suppressed in HCC and PDAC [27, 28]. Concomitantly, BCKDK expression was increased in HCC, however, more distal enzymes of BCAA catabolism, ACADS, and ACADSB were downregulated [27]. Our data are in agreement with these findings demonstrating dysregulated BCAA catabolic enzyme expression in MDA-MB231 and BT549 cells, highlighting the involvement of BCAA catabolizing enzymes and metabolites in TNBC tumorigenesis.

Two studies have recently identified different phosphorylation sites on BCKDK that mediates cancer proliferation, signaling and metastasis [12, 13]. Notably, stabilizing BCKDK via Src promoted migration, invasion, and metastasis in colorectal cancers [12]. Our data in TNBCs demonstrate that inhibiting BCKDK impairs TNBC proliferation and sensitizes TNBCs to DOX toxicity. We propose that the metabolic effects of BCKDK are plausibly an outcome of altered BCAA–BCKA flux towards oxidation and away from protein synthesis. Indeed, increased intracellular BCAA accumulation upon BCKDHA deletion resulted in increased cell proliferation in an immortalized hepatocyte cell line [27]. Moreover, increased extracellular secretion of BCKAs in a BCAT1 dependent manner supports cell proliferation in PDACs [8] and survival of glioblastoma cells by evading immune surveillance [9]. Our study also demonstrated that BCKDK inhibition was sufficient to reduce intracellular and secreted BCKAs in MDA-MB231 cells, likely contributing to reduced cell proliferation. Since TNBCs are classically treated with combination therapy involving anthracyclines [2], such as DOX, targeting BCKDK might trigger a metabolic vulnerability rendering TNBCs susceptible to DOX toxicity.

Prior reports showed that BCKDK function as a regulatory, upstream kinase of MEK [31] and ERK1/2 [13], drivers of cell proliferation. Indeed, BCKDK stabilization is an alternative mechanism to activate RAS/RAF/MEK/ERK signaling and confer drug resistance [31]. Whether the accumulation of BCAA metabolites, such as BCKAs, activates oncogenes and drives growth factor signaling in TNBCs warrants investigation. We questioned whether BCKDK silencing with concomitant DOX treatment potentiated BCKA oxidation, reducing intracellular BCKAs, compromising growth and increasing cell death. In our study, we observed that when TNBCs were exposed to DOX, BCKDK expression and intracellular BCKAs levels were downregulated. Moreover, silencing BCKDK exacerbated cytotoxic effects of DOX in a caspase 3/7 dependent manner decreasing cell proliferation. Although the mechanism by which BCKDK silencing triggers apoptosis induction remains unclear, we theorize that perturbations in protein synthesis and altered mitochondrial metabolism might precede or follow apoptotic events and/or increased BCKA oxidation following BCKDK inhibition.

Dysregulation of protein translation and anomalous energy metabolism are characteristic of cancers, processes that are sensitive to the effects of chemotherapeutic agents [32, 33]. Chemotherapeutics such as DOX can accumulate within the mitochondria resulting in mitochondrial dysfunction and energy stress by disrupting the electron transport chain (ETC) and reducing mitochondrial oxidative capacity [19]. Indeed, a decline in ATP synthesis is associated with mitochondrial accumulation of DOX [34]. We found that BCKDK inhibition reduced ATP production and oxygen consumption in MDA-MB231 cells but did not exacerbate DOX-mediated suppression of respiration or energy production. Notably, BCKDK deficient fibroblasts show reduced intracellular ATP and respiration, with a corresponding increase in ROS production and mitochondrial hyperfusion, which was not observed following BCKDK silencing in BCKDH deficient fibroblasts from MSUD patients [35]. Interestingly, silencing BCKDHA in PDACs did not affect mitochondrial metabolism or oxygen consumption [26], suggesting a direct involvement of accelerated BCAAs catabolism in mitochondrial dysfunction. Moreover, our transcriptomics data in BCKDK depleted cells identified reduced expression of several mitochondrial genes associated with complex formation, glycine metabolism, and cholesterol trafficking. However, it remains to be determined how and why targeting BCKDK in TNBCs impairs mitochondrial function due to the increased BCKA oxidation burden.

Changes in the metabolic microenvironment within TNBCs hyperactivates signaling pathways of protein translation and biosynthesis, enabling uncontrolled growth and survival. Protein synthetic rates are significantly reduced following apoptosis induction, where caspase-dependent proteolytic cleavage and altered phosphorylation states of translation initiation factors (eIF4GI, eIF4GII, eIF4E, eIF4B, and eIF2α) influence cancer progression [21, 36]. We found that BCKDK inhibition decreased protein synthesis. Consistent with this finding, we also observed a significant increase in inhibitory phosphorylation of eEF2 at Thr 56 upon BCKDK depletion. eEF2 phosphorylation impedes translocation of peptidyl-tRNA from the A site to the P site on the ribosome. High intracellular Ca^2+^ levels increase phosphorylation of eEF2 [37]. Our transcriptomic data in BCKDK depleted cells revealed enrichment of the GO-term related to NFAT-calcineurin signaling cascade, which in turn is activated by high intracellular Ca^2+^ levels, a likely trigger for increased eEF2 phosphorylation. eEF2 is downstream of the AMPK-mTORC1 axis where it is inhibited by AMPK and activated by mTORC1. Our data in both TNBC cell types showed impaired mTORC1 signaling upon BCKDK inhibition and significant upregulation of SESN2, the negative regulator of mTORC1. The suppressed activation of mTORC1 signaling explains the inactivation of eEF2. In addition, reduction in protein synthesis following BCKDK inhibition was associated with increased inhibitory phosphorylation of eIF2α at Ser 51. eIF2α phosphorylation increases in response to stress and cancer [36]. eIF2α phosphorylation at Ser 51 inhibits the eIF4B catalyzed guanine nucleotide exchange function of eIF2, preventing the formation of the 43S preinitiation complex [21]. It is unclear whether a decline in mTORC1 driven protein synthesis induces apoptosis [38] or increased apoptosis suppresses global translation or a diminished amino acid availability in TNBCs with inhibited BCKDK.

In conclusion, our study highlights BCKDK as a target for potentiating cytotoxic effects of DOX in TNBCs and DOX’s impact on likely accelerating BCAA catabolism. This study demonstrates that BCKDK inhibition alone is sufficient to induce caspase activation, ATP insufficiency, and decrease protein translation, rendering TNBCs susceptible to cell death. The metabolic fate of accumulating BCKAs following BCKDK silencing is either to get reaminated to BCAAs or be oxidized. Since BCAAs (majorly leucine) is recognized as a critical amino acid to activate mTOR, the reaminative fate for BCKAs is unlikely in our study, as mTOR signaling is suppressed. However, changes in mTOR signaling may or may not be dependent on changes in BCAA levels. Further studies are required to elucidate if the susceptibility of TNBC to loss of BCKDK function is an outcome of increased BCKAs oxidation or altered availability of BCKAs for signaling and metabolic processes. Furthermore, mechanistic understanding is warranted on how BCKDK silencing triggers caspases and apoptotic pathways and if p53 function mediates these effects. DOX’s genotoxic stress also involves DNA intercalation and double-strand breaks; whether BCKDK silencing sensitizes cells to DOX-induced DNA damage, remains to be explored. In addition, clarity on the molecular events driving increased clearance of intracellular BCKAs in DOX treated TNBC is paramount with a focus on BCKDK interactome or upstream regulators, such as the transcriptional factor, KLF15. Since BCKDK was recently identified as a cytosolic kinase regulating hepatic lipid accumulation [39], future studies will also interrogate metabolic pathways that co-operate with BCAA metabolism to promote tumor growth or compensate for sustaining cell proliferation when BCAA metabolism is inhibited.

## Materials and methods

### Cell lines and culture conditions

MCF10A cells (CRL-10317, US) and BT549 cells (HTB-122) were obtained from ATCC. MDA-MB231 cells were a gift from Dr. G. Robichaud (Université de Moncton). The cells were cultured according to our previously published studies [3]. All the cell lines were maintained at 37 °C in a humidified atmosphere of 5% CO_2_. The cell lines have not been genetically authenticated. For the BT2 experiments, cells were pretreated for 20 h with the desired concentration of BT2 (Sigma, A760956 and Matrix Scientific, 028302). BT2 from Sigma was dissolved in cremaphore solution, generously gifted by Ayappan Subbiah (Sevengenes Inc.).

### Transfections and viral transductions

Adenoviral vector expressing BCKDK shRNA (shADV-225358) and the control vector for scrambled shRNA GFP (Cat# 1122) were obtained from Vector Biolabs (USA). Adenoviral infection of cells was done 24 h post-plating, and the multiplicity of infection was kept constant between the control and experimental constructs. siRNA knockdown of BCKDK was performed using Ambion silencer select siRNA oligonucleotides (Thermo-Fisher Scientific Cat# 4390824). The siRNAs used in this paper were siBCKDK#1: #s20126, siBCKDK#2: #s20127, siRNA negative control (Cat# 4390844). Transfection was achieved using Lipofectamine RNAiMAX (Invitrogen) following the manufacturer’s instructions with a concentration of 10 nM of siRNA per plate.

### Cell lysate processing and immunoblotting

Cell pellets were sonicated in ice-cold lysis buffer (containing 20 mM Tris-HCl, pH 7.4, 5 mM EDTA, 10 mM Na_4_P_2_O_7_ (Calbiochem), 100 mM NaF, 1% Nonidet P-40, 2 mM Na_3_VO_4_, protease inhibitor (10 ml/ml; Sigma), and phosphatase inhibitor (10 ml/ml; Calbiochem) and centrifuged at 16,000*g* for 15 min. Protein concentration determination, immunoblotting and development of immunoblots were performed as described previously [40]. The list of primary antibodies is described in Table S1. Densitometric analysis was performed using Image laboratory software (Bio-Rad), and the quantifications were normalized by total protein loading using GraphPad software (Clarivate).

### qPCR analysis

mRNA levels of BCAA-catabolizing enzymes were determined using qPCR by employing validated optimal reference gene pairs. Primer information of the target and reference genes is provided in Table S2. RNA isolation, quality control, cDNA synthesis, and qPCR analysis were performed as described previously [41].

### Trypan blue exclusion and metabolic activity assays

For cell viability, 1 × 10^4^ cells were plated in 24-well plates in triplicates. The total number of viable cells was calculated using the trypan blue exclusion assay. For measuring metabolic activity, cells were plated in 96-well plates and treated with DOX for 18 h followed by incubated in drug-free media for 48 h. The metabolic activity of viable cells were measured by incubating the cells with Presto Blue (Thermo-Fischer Scientific, A13262) for 3 h at 37 °C. Fluorescent intensity was read at Ex/Em = 560/590 on a microplate fluorometer (Synergy H4). Cell Counting Kit-8 (CCK-8; Sigma Aldrich, 96992) was used to measure cell viability and metabolic activity according to the manufacturer’s protocol. Briefly, 1 × 10^5^ MDA-MB231 cells were seeded in 96-well plates transfected with the siRNAs for different time points followed by DOX treatment for 18 h. Following the treatments, the cells were incubated in CCK-8 solution at 37 °C for 4 h. Synergy H4 microplate reader was used to measure the absorbance at 450 nm. 

### Extracellular flux analyzer studies

Mitostress assay was performed according to Khan et al. [42] using the Seahorse XFe24 analyzer (Agilent Technologies Inc. CA, USA). MDA-MB231 or MCF10A cells were plated at a density of 30,000 cells/per well. Briefly, cells were incubated with XF Assay medium (with 20 mM glucose, 1 mM sodium pyruvate, and 1 mM glutamate, without sodium bicarbonate) for 1 h. One micrometre of oligomycin, 1 μM of FCCP [carbonyl cyanide‐4‐(trifluoromethoxy)phenylhydrazone], and 1 μM each of rotenone and antimycin A was injected over 100 min to measure the different stages of respiration. The assay was normalized with protein and analyzed with the XFe 2.0.0 software (Agilent Technologies Inc. CA, USA).

### Apoptosis array

The expression of 43 apoptosis-associated proteins in duplicates was determined by the Human apoptosis antibody array (Abcam, ab134001). Membranes were incubated with 250 μg of cell extracts, immunoblotted, and developed using the chemidoc (BioRad) according to manufacturers’ instructions. Densitometric analysis was performed using Image laboratory software (Bio-Rad).

### LDH cytotoxicity assay

LDH cytotoxicity assay was performed using the assay kit (Invitrogen, C20301) according to the manufacturer’s instructions. Briefly, 1 × 10^4^ cells were plated in a 96-well plate for experimental conditions as well as spontaneous and maximum LDH activity controls. On the day of the assay, 10 μl of 10× lysis buffer was added to wells serving as the maximum LDH activity controls and incubated at 37 °C for 45 min. The reaction mixture was added to the collected media in a 1:1 ratio and incubated at RT for 30 min in the dark, followed by adding a stop solution. The absorbance was measured at 490 nm, and 680 nm and the assay was normalized by protein concentration.

### Caspase 3 activity assay

Caspase activity assay (Caspase-3 Activity Assay Kit, Cell Signaling Technology, 5723) was performed according the manufacturer’s instructions. Briefly, 1 × 10^4^ cells were plated in a 96-well plate and were harvested using ice-cold lysis buffer. Lysates from two wells within the same treatment group were combined for all treatment groups and transferred to a black plate. Fluorescence was measured immediately at Ex/Em 380/440 for the 0 h reading. A second reading was taken after 1 h incubation in the dark at 37 °C. The assay was normalized by protein concentration.

### AHA incorporation assay

AHA incorporation analysis was performed using the Click-iT AHA Alexa Fluor 488 Imaging Kit (Thermo-Fisher Scientific, C10289) according to the manufacturer’s instructions. Briefly, cells on coverslips were pulse-labeled with AHA for 30 min in methionine/cysteine free DMEM media without serum before performing the click labeling. Coverslips were mounted in Prolong anti-fade mounting media (Thermo-Fisher Scientific, P36930) and imaged with a Zeiss Axio Observer Z1 equipped with an Apotome.2 structural illumination units using a 20× Plan-Apochromat objective (NA: 0.8, air). Images were processed for analysis in ImageJ, and image processing was identical for each image set. Images were analyzed in Cell Profiler, and data are represented as the mean ± S.D. per cell of three experiments.

### RNA-seq analysis and bioinformatics

MDA-MB231 cells were transfected with either of two siRNA’s targeting BCKDK or a scrambled siRNA and cultured for 48 h before cells were harvested and RNA extracted using the Qiagen RNeasy mini kit according to the manufacturer’s instructions. RNA-Seq was conducted by McGill University and the Genome Quebec Innovation Center (Montreal, Canada) with the Illumina NovaSeq 6000 S2 PE100—50 M platform. Analysis of the transcriptomic data was performed according to our previous published study [3]. Testing for differential expression was conducted only on genes with an average estimated count of greater than 0.3. Genes were considered significantly differentially expressed if the adjusted P-value was less than 0.01 for both siBCKDK#1 and siBCKDK#2 groups, and the fold change was occurring in the same direction. RNA-Seq data are deposited in NCBI’s Gene Expression Omnibus under the assecession number GSE163297.

### SunSET method

Protein synthesis was measured in vitro by the SunSET assay as described previously [40]. Briefly, BT549 cells were either transfected with siBCKDK #1 or #2 and siCon for 48 h or with 500 µM BT2 for 20 h followed by treatment with 1 µM puromycin dihydrochloride (Sigma, P8833) for 30 min followed by a 30 min chase with complete media. Puromycin incorporation was detected by Western blotting using the monoclonal puromycin antibody.

### BCKA measurements

For BCKA measurements, cells were incubated with serum-free DMEM low glucose, leucine-free medium (Sigma, D9443) for 24 h or during the duration of the treatment.

#### Secreted BCKA extraction

Twenty microlitres of the media and 120 µl of internal standard (4 mg/ml in H_2_O) containing leucine-d3 (CDN Isotopes), 40 µl of MilliQ water, 60 µl of 4 M perchloric acid (VWR) were combined and vortexed. Proteins were precipitated in two sequential steps, followed by centrifugation at 13,000 rpm for 15 min at 4 °C. Supernatants collected from both steps were combined for measuring BCKAs.

Intracellular BCKA extraction, BCKA derivatization, and quantification were done according to our previously published study [40].

### Statistical analysis

Data are expressed as mean ± S.D. unless otherwise indicated. Statistical analyses were conducted using Prism software (GraphPad, La Jolla, CA, USA). Comparisons between multiple groups were performed using one-way or two-way analysis of variance followed by a Tukey post hoc test, as appropriate. Data sets with two groups were analyzed using a two-tailed student’s *t* test. *p* Values of 0.05 were considered statistically significant.

## Supplementary information

Figure S1

Figure S2

Figure S3

Figure S4

Figure S5

Figure S6

Supplementary Data-Legends and Tables

Author Contribution form

Authorship Addition Justification Approval Form

## Data Availability

All data and materials used in the current study are available from the corresponding author upon request. The datasets generated and/or analyzed during the current study are available in NCBI’s Gene Expression Omnibus. Accession ID: GSE163297.
