# Supplementary figures and images for: Inhibiting BCKDK in triple negative breast cancer suppresses protein translation, impairs mitochondrial function, and potentiates doxorubicin cytotoxicity
Source: Cell Death Discov. 2021 Sep 15;7:241. doi: 10.1038/s41420-021-00602-0 (PMC8443725; doi:10.1038/s41420-021-00602-0)

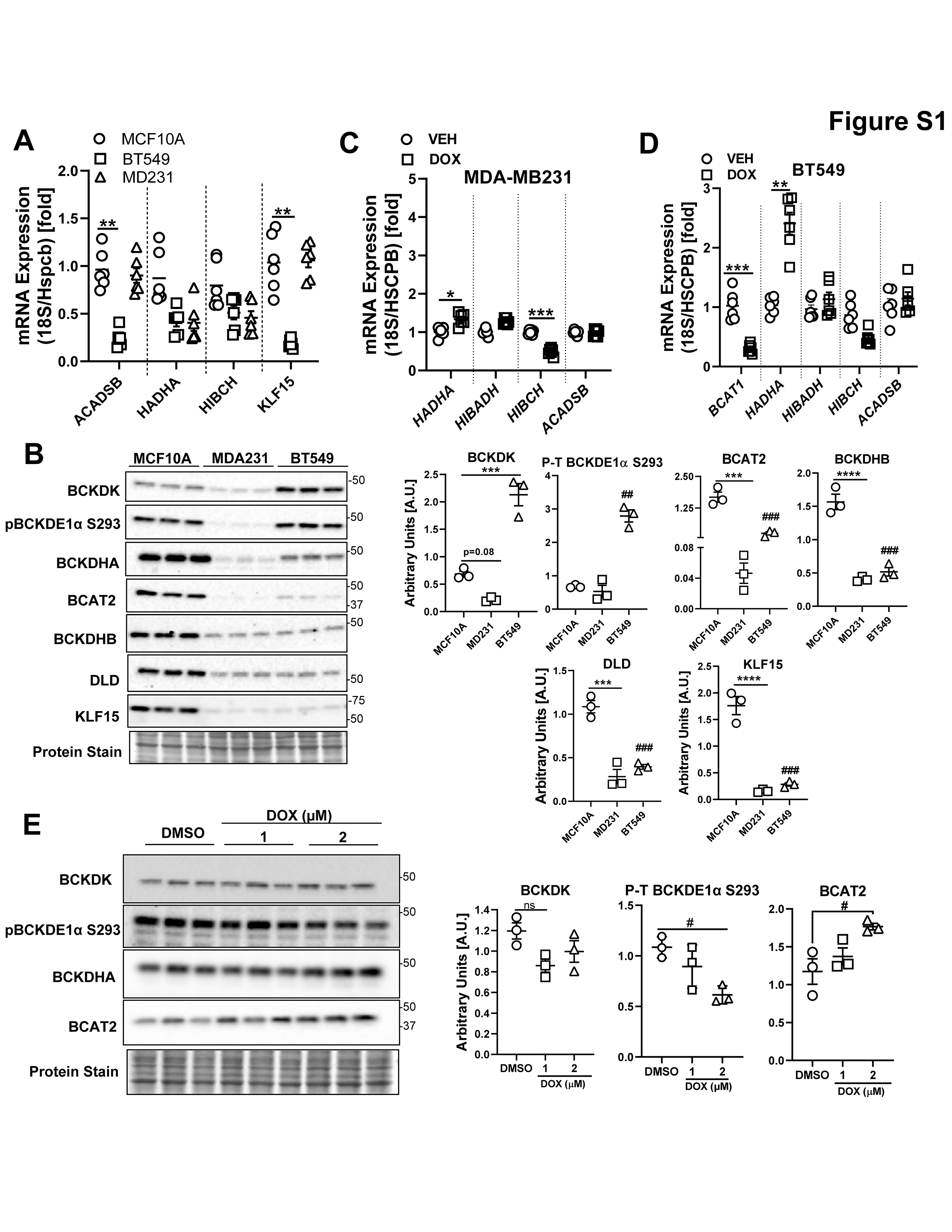

Supplement: Supplementary file 1 — Figure S1 [file 41420_2021_602_MOESM1_ESM.png]

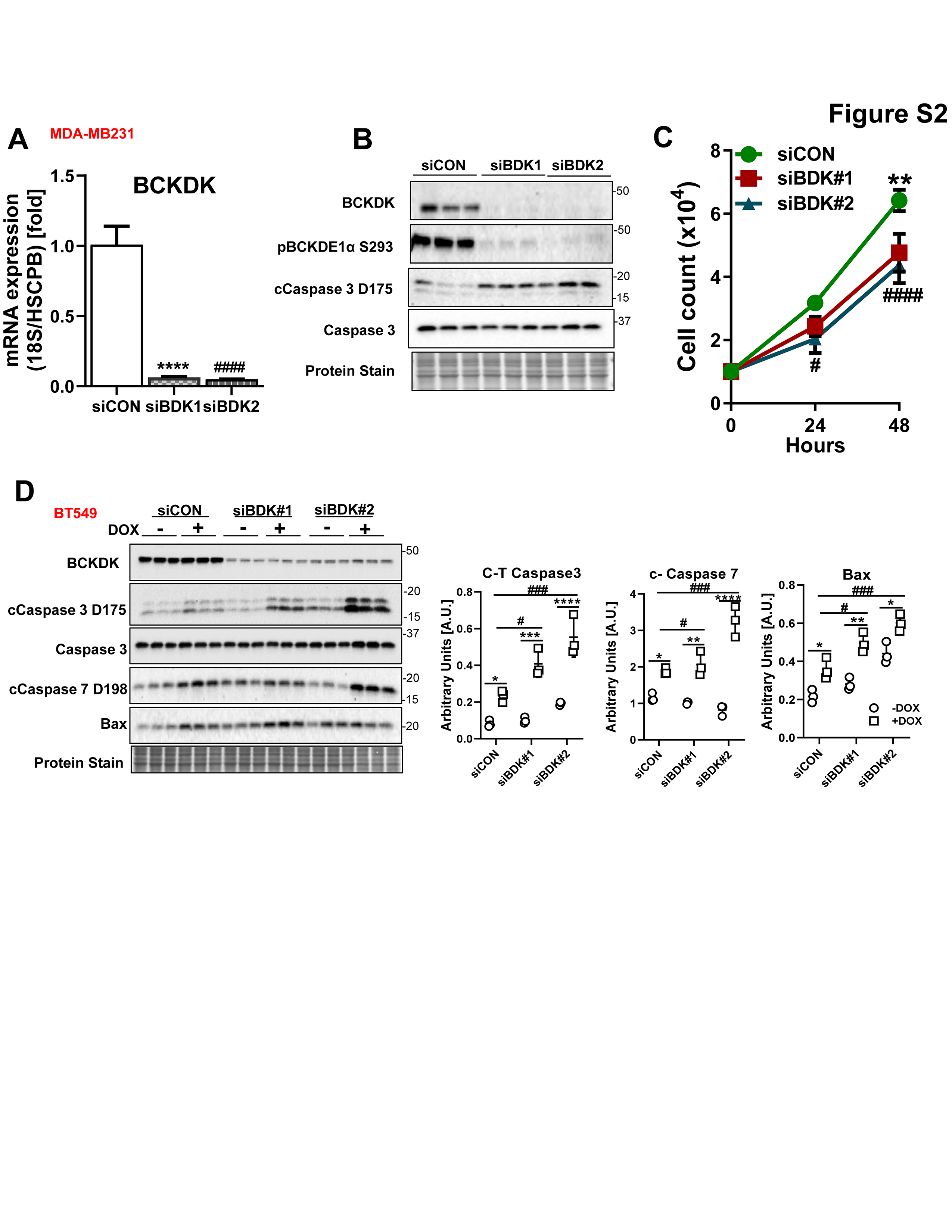

Supplement: Supplementary file 2 — Figure S2 [file 41420_2021_602_MOESM2_ESM.png]

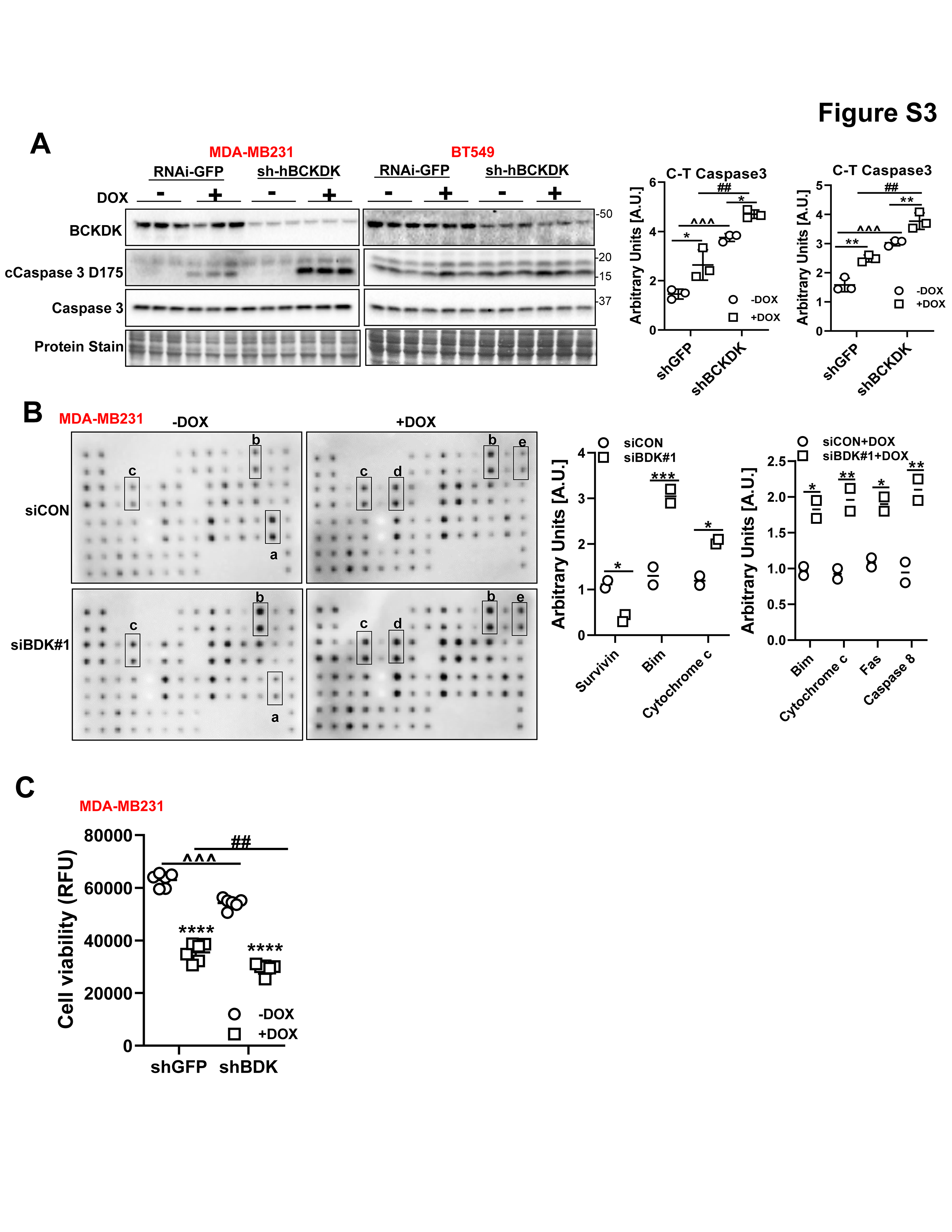

Supplement: Supplementary file 3 — Figure S3 [file 41420_2021_602_MOESM3_ESM.png]

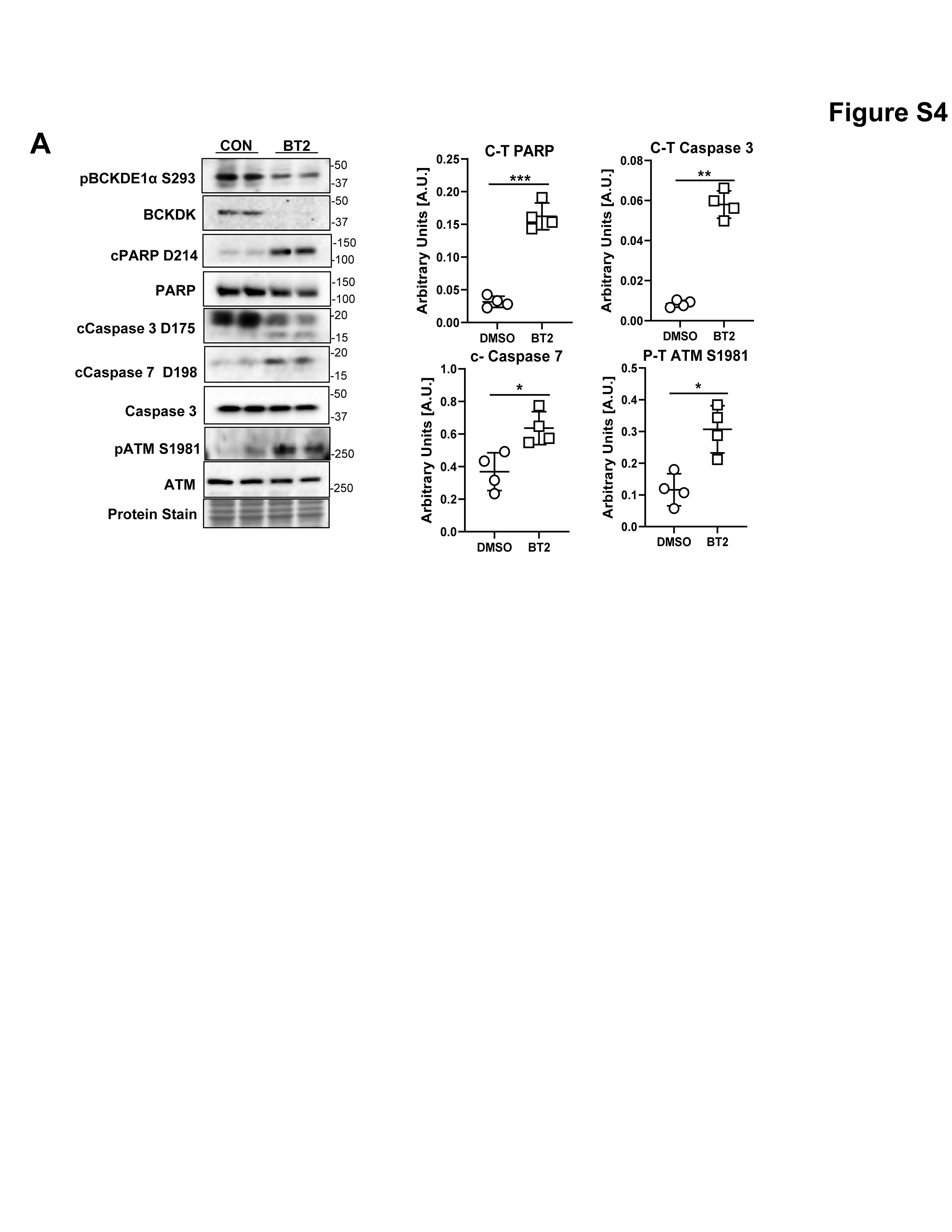

Supplement: Supplementary file 4 — Figure S4 [file 41420_2021_602_MOESM4_ESM.png]

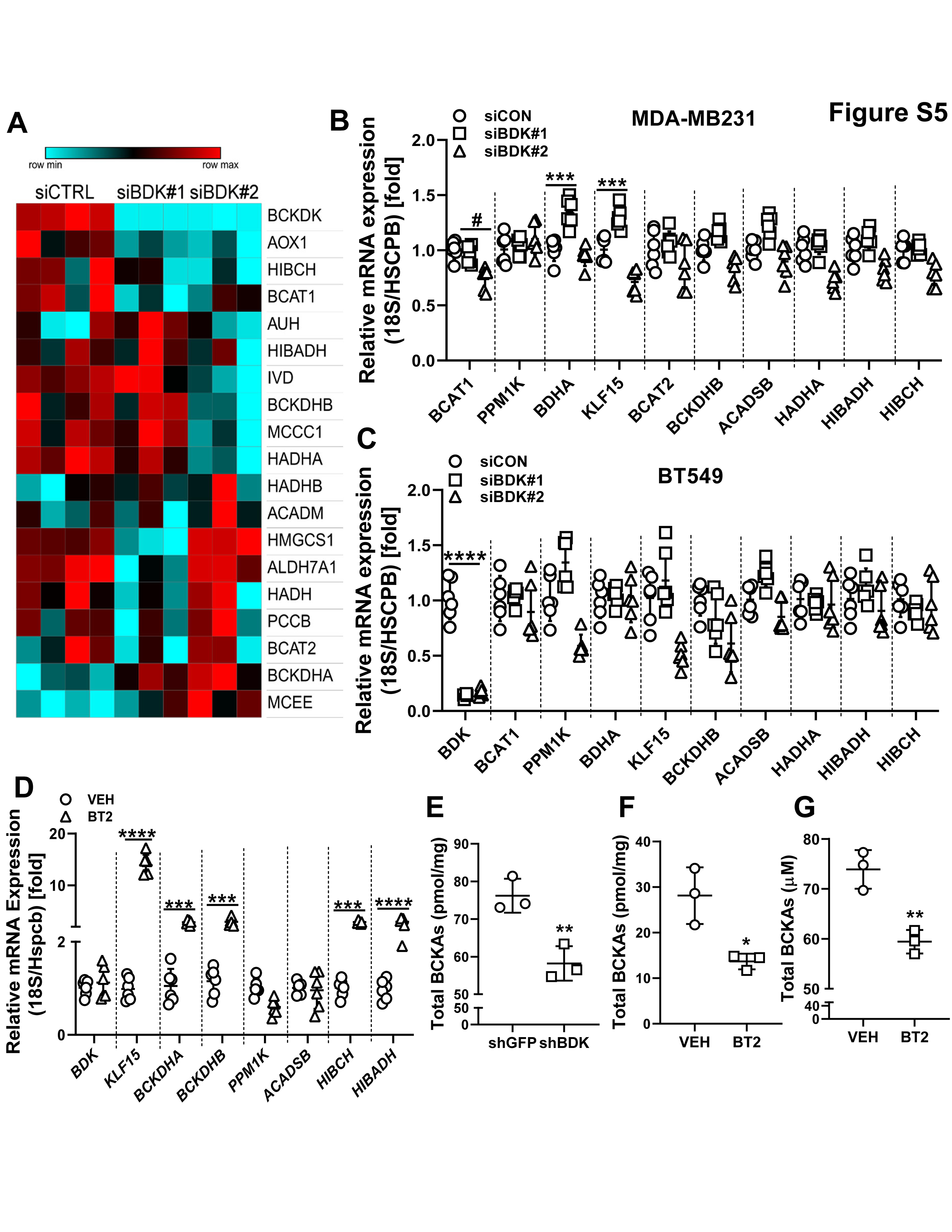

Supplement: Supplementary file 5 — Figure S5 [file 41420_2021_602_MOESM5_ESM.png]

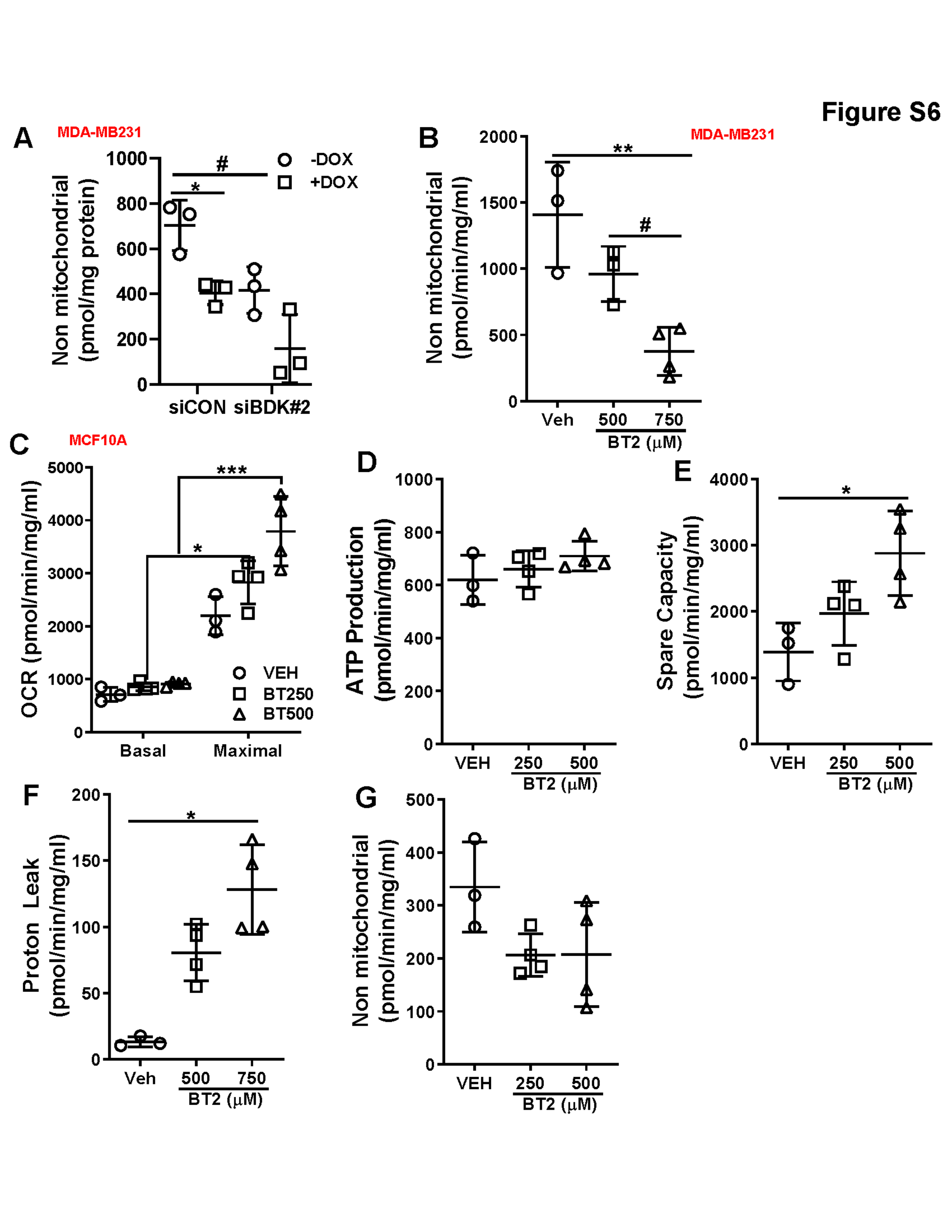

Supplement: Supplementary file 6 — Figure S6 [file 41420_2021_602_MOESM6_ESM.png]
