# Supplementary material for: Inhibiting BCKDK in triple negative breast cancer suppresses protein translation, impairs mitochondrial function, and potentiates doxorubicin cytotoxicity
Source: Cell Death Discov. 2021 Sep 15;7:241. doi: 10.1038/s41420-021-00602-0 (PMC8443725; doi:10.1038/s41420-021-00602-0)
Supplement: Supplementary file 7 — Supplementary Data-Legends and Tables [file 41420_2021_602_MOESM7_ESM.docx]

**Inhibiting BCKDK in triple negative breast cancer suppress protein translation, impair mitochondrial function, and potentiate doxorubicin cytotoxicity**

**Running Title:** *Inhibiting BCKDK increase TNBC DOX toxicity*

Dipsikha Biswas^1^, Logan Slade^1^, Luke Duffley^1^, Neil Mueller^1^, Khoi Thien Dao^1^, Angella Mercer^1^, Shanmugasundaram Pakkiriswami^1^, Yassine El Hiani^2^, Petra Kienesberger^1^ and Thomas Pulinilkunnil^1*^

**SUPPLEMENTARY INFORMATION**

**Supplementary figure legends:**

**Figure S1. BCAA degradation enzyme expression is suppressed in TNBCs and can be revived by DOX treatment.** A) Quantification of *ACADSB*, *HADHA*, *HIBCH*, and *KLF15* mRNA expression corrected to 18S/HSPCB reference genes in MCF10A, BT549 and MDA-MB231 cells. B) Immunoblot and densitometric analysis of BCKDK, total and phosphorylated BCKDHA E1α Ser 293, BCAT2, BCKDHB, DLD and KLF15 in MCF10A, MDA-MB231 and BT549 cells. C-D) BCAA catabolic enzyme expression in TNBCs treated with 2µM DOX for 18h. Quantification of *BCAT1*, *ACADSB*, *HADHA*, *HIBCH*, mRNA expression corrected to 18S/HSPCB reference genes in MDA-MB231 (C) and BT549 (D) cells. E) Immunoblot and densitometric analysis of BCKDK, total and phosphorylated BCKDHA E1α Ser 293 and BCAT2 in BT549 cells treated with 1µM and 2µM DOX for 18h. Data presented as mean ± S.D. Statistical analysis was performed using a two-way ANOVA followed by a Tukey’s multiple comparison test; *p <0.05, **p < 0.01, **** p <0.0001 as indicated.

**Figure S2. BCKDK knockdown increases cell death and potentiates DOX’s effects in TNBCs.**  mRNA quantification of *BCKDK* (A) and protein expression (B) of BCKDK and cleaved and total caspase 3 expression in MDA-MB231 cells transfected with siRNA targeting BCKDK at exon 4 (siBDK#1) and exon 5 (siBDK#2). C) Cell counts at 0, 24 and 48h of MDA-MB231 cells transfected with siCON, siBDK#1 or siBDK#2. D) Immunoblot and densitometric analysis of BCKDK, total and cleaved Caspase 3, cleaved Caspase 7, total and Bax in BT549 cells transfected with siCON, siBDK#1 or siBDK#2 for 72h followed by 2µM DOX or DMSO for 18h. Data presented as mean ± S.D. Statistical analysis was performed using a two-way ANOVA followed by a Tukey’s multiple comparison test; *p <0.05, **p < 0.01, **** p <0.0001 as indicated.

**Figure S3. BCKDK silencing induces genes associated with cell death in TNBCs.** A) Immunoblot and densitometric analysis of total and cleaved Caspase 3 in MDA-MB231 and BT549 cells transduced with either shGFP or shBCKDK for 48h followed by 2µM DOX or DMSO for 18h. G) Metabolic viability of MDA-MB231 cells transduced with either shGFP or shBCKDK and treated with 2µM DOX or DMSO for 18 h plus 48 h in drug-free medium. C) Antibody-immobilized PVDF membranes containing 43 different apoptosis associated proteins incubated with extracts of CON (siCON) and BCKDK (siBDK#1) silenced MDA-MB231 cells, treated with or without 2µM DOX for 18h. Differentially expressed proteins (fold change more than 1.5 or less than 0.6) are quantified and indicated on the blots. a=Survivin, b=Bim, c=Cytochrome, d= Fas, e= Caspase8.

**Figure S4. BT2 at high doses induce cell death markers in TNBCs.** A) Immunoblot and densitometric analysis of phosphorylated BCKDE1α Ser 293, BCKDK, total and cleaved Caspase 3, cleaved Caspase 7, total and cleaved PARP, total and phosphorylated ATM Ser 1981 in MDA-MB231 cells treated with 500µM BT2 for 20h. Densitometric analysis is from three independent experiments.

**Figure S5. BT2 augments mRNA expression of BCAA catabolic genes and reduces BCKA accumulation and secretion.** A) Heatmap for the genes involved in BCAA catabolism which were differentially regulated by BCKDK knockdown. B-C) Quantification of BCKDK, BCKDHA, PPM1K, BCKDHB, BCAT2, BCAT1, ACADSB, HADHA, HIBCH, HIBADH and KLF15 mRNA expression corrected to 18S/HSPCB reference genes in B) MDA-MB231 and C) BT549 cells transfected with siCON, siBDK#1 or siBDK#2 for 72h. D) Quantification of BCKDK, BCKDHA, PPM1K, BCKDHB, ACADSB, HIBCH, HIBADH and KLF15 mRNA expression corrected to 18S/HSPCB reference genes in BT549 cells treated with 500µM BT2 for 20h. Statistical analysis was performed using a two-way ANOVA followed by a Tukey’s multiple comparison test; *p <0.05, **p < 0.01, **** p <0.0001 as indicated. E) UPLC MS/MS analysis of intracellular BCKAs in MDA-MB231 cells transduced with shBCKDK and shGFP for 48h. Measurement of intracellular (F) and secreted BCKAs in the media (G) by UPLC MS/MS in MDA-MB231 cells treated with 500µM BT2 for 20h. Data presented as mean ± S.D. Statistical analysis was performed using a Student’s t-test; *p <0.05, **p < 0.01, **** p <0.0001 as indicated.

**Figure S6. BT2 does not affect mitochondrial respiration in MCF10A cells.** A-B) Non mitochondrial respiration measured in MDA-MB231 cells A) transfected with siCON or siBDK#2 for 48h or B) treated with 250µM or 500µM BT2 for 20h, in the presence of 25mM glucose. C-G) Basal and maximal OCR (C), ATP production (D), spare capacity (E), proton leak (F) and non mitochondrial respiration (G) measured in MCF10A cells treated with 250µM or 500µM BT2 for 20h in the presence of 25mM glucose. Data presented as mean ± S.D. Statistical analysis was performed using a was performed using two-way ANOVA followed by a Tukey’s multiple comparison test for (A) or Student’s t-test for (B); *p <0.05, **p < 0.01, **** p <0.0001 as indicated.

**Table S1.** List of antibodies.

(Fine chemicals, if noted otherwise under the Materials and Methods segment, are from Sigma.)

| **Antibodies** | **Source** | **Identifier** |
| --- | --- | --- |
| BCKDK | My Biosource | MBS 275719 |
| p-BCKDE1α S293 | Bethyl Lab | A303-567A |
| BCKDHA | My Biosource | MBS 275832 |
| BCKDHB | Santa Cruz Biotechnology Inc. | H-6; sc-374630 |
| BCAT2 | Invitrogen | PA5-21549 |
| DLD | Santa Cruz Biotechnology Inc. | G-2; sc-365977 |
| KLF15 | Novus Biologicals | NBP2-24635 |
| cCaspase 3 D175 | Cell Signaling Technologies | 9664 |
| cCaspase 7 D198 | Cell Signaling Technologies | 8438 |
| Caspase 3 | Cell Signaling Technologies | 9662 |
| cPARP D124 | Abcam | ab110315 |
| PARP | Cell Signaling Technologies | 9532 |
| p-ATM S1981 | Cell Signaling Technologies | 5883 |
| ATM | Cell Signaling Technologies | 2873 |
| Sestrin 2 | Cell Signaling Technologies | 8487S |
| p-mTOR S2448 | Cell Signaling Technologies | 5536 |
| mTORC1 | Cell Signaling Technologies | 2972 |
| p-P70S6K T389 | Cell Signaling Technologies | 9234 |
| P70S6K | Cell Signaling Technologies | 2708 |
| p-S6 S240/244 | Cell Signaling Technologies | 5364 |
| S6 | Cell Signaling Technologies | 2217 |
| p-eEF2 T56 | Cell Signaling Technologies | 2331 |
| eEF2 | Cell Signaling Technologies | 2332 |
| p-eEF2K S366 | Cell Signaling Technologies | 3691 |
| eEF2K | Cell Signaling Technologies | 3692 |
| p-eIF2α S51 | Cell Signaling Technologies | 9721 |
| eIF2α | Cell Signaling Technologies | 9722 |
| Puromycin clone 12D10 | Millipore-Sigma | MABE343 |
| Total OXPHOS cocktail | Abcam | ab110413 |
| mouse anti-rabbit IgG-HRP | Santa Cruz Biotechnology Inc. | sc-2357 |
| m-IgG kappa BP-HRP | Santa Cruz Biotechnology Inc. | sc-516102 |

**Table S2.** List of primers.

| **Primer** | **Gene name** | **Sequence 5'-3'** |
| --- | --- | --- |
| h- BCKDK-F  h- BCKDK-R | Branched chain ketoacid dehydrogenase kinase | GACTTCCCTCCGATCAAGGAC  CTCTCACGTAGGCCCTCTG |
| h-BCKDHA-F  h-BCKDHA-R | Branched chain keto acid dehydrogenase E1 alpha polypeptide | CTACAAGAGCATGACACTGCTT  CCCTCCTCACCATAGTTGGTC |
| h- BCKDHB-F  h- BCKDHB-R | Branched chain keto acid dehydrogenase E1 subunit beta | TGGAGTCTTTAGATGCACTGTTG  CGCAATTCCGATTCCAAATCCAA |
| h-KLF15 -F  h- KLF15 -R | Kruppel like factor 15 | CGGCTGGAGGTTCTCGCGCTCTG AGGCTGGGGTTCAGGGCGCTTTC |
| h-PPM1K-F  h-PPM1K-R | Protein phosphatase Mg2+/Mn2+ dependent 1K | ATAACCGCATTGATGAGCCAA CGCACCCCACATTTTCCAAG |
| h-BCAT2-F  h-BCAT2-R | Branched chain amino acid transaminase 2 | CGCTCCTGTTCGTCATTCTCT CCCACCTAACTTGTAGTTGCC |
| h-ACADSB-F h-ACADSB-R | Acyl-CoA dehydrogenase short branched chain | GATGGCAAATGTAGACCCTACC GGAGGTTTTAGTCCTGGTCCC |
| h-HADHA-F  h-HADHA-R | Hydroxyacyl-CoA dehydrogenase trifunctional multienzyme complex subunit alpha | CTGCCCAAAATGGTGGGTGT GGAGGTTTTAGTCCTGGTCCC |
| h-HIBCH-F  h-HIBCH-R | 3-hydroxyisobutyryl-CoA hydrolase | TGGTTCTTGCCAGAAACCTTATG  GTAGCCACTCGAAATTGCCCA |
| h-BCAT1-F  h-BCAT1-R | Branched chain amino acid transaminase 1 | AGCCCTGCTCTTTGTACTCTT  CCAGGCTCTTACATACTTGGGA |
| h-HIBADH-F  h-HIBADH-R | 3-hydroxyisobutyrate dehydrogenase | TGCTGCCCACCAGTATCAATG  GCAGGATCAATAGTGCTGGAATC |
| h-18S-F  h-18S-R | 18S ribosomal RNA | AGAAACGGCTACCACATCCA  CACCAGACTTGCCCTCCA |
| h-HSP90AB1-F  h-HSP90AB1-R | Heat shock protein HSP 90-beta | TCTGGGTATCGGAAAGCAAGCC  GTGCACTTCCTCAGGCATCTTG |
