# Supplementary material for: Inhibiting BCKDK in triple negative breast cancer suppresses protein translation, impairs mitochondrial function, and potentiates doxorubicin cytotoxicity
Source: Cell Death Discov. 2021 Sep 15;7:241. doi: 10.1038/s41420-021-00602-0 (PMC8443725; doi:10.1038/s41420-021-00602-0)
Supplement: Supplementary file 8 — Author Contribution form [file 41420_2021_602_MOESM8_ESM.pdf]

DECLARATION OF CONTRIBUTIONS TO ARTICLE

ADMC

Manuscript Number: CDDISCOVERY-21-1626

Journal Name: *Cell Death Discovery* (the 'Journal')

Proposed Title of the Contribution:

Inhibiting BCKDK in TNBCs increase cytotoxicity, suppress protein translation and mitochondrial function, and potentiate doxorubicin cytotoxicity (the 'Contribution)

Author(s):

Dipsikha Biswas, Logan Slade, Luke Duffley, Neil Mueller, Khoi Thien Dao, Angella Mercer, Sundaram Pakkiriswami, Yassine El Hiani, Petra Kienesberger and Thomas Pulinilkunnil (the 'Authors')

For all *CDDis* articles, each person named as an author in the published version must be able to show he or she has contributed substantially to the article.

Authorship credit should be based on 1) substantial contributions to conception and design, acquisition of data, or analysis and interpretation of data; 2) drafting the article or revising it critically for important intellectual content; and 3) final approval of the version to be published. Authors should meet conditions 1, 2 and 3.

Any person who cannot be shown to have made a substantial contribution to the article cannot be listed as an author in the final version. The name of any person who is deemed to have made a minor contribution can, however, appear in the Acknowledgments section of the article.

Please complete the table below to indicate the contributions of all named authors to the manuscript.

Author Full Name:	Specification of Contribution to the Manuscript:
Dipsikha Biswas	Conceptualization, investigation, data curation, analysis, methodology, writing original draft, review and editing
Logan Slade	RNA sequencing analysis, cell culture, review and editing of final draft
Luke Duffley	Investigation
Neil Mueller	Investigation
Khoi Thien Dao	Investigation
Angella Mercer	UPLC analysis
Sundaram Pakkiriswami	RNA sequencing analysis
Yassine El Hiani	Resources, review and editing of final draft
Petra Kienesberger	Resources, review and editing of final draft
Thomas Pulinilkunnil	Conceptualization, resources, supervision, review and editing, funding acquisition

Please complete the table below to indicate the contributions of all named authors to the figures.

Figure 1: D.B., N.M. and A.M investigation, formal analysis, D.B. and T.P. conceptualization, T.P. resources, D.B. software, methodology and validation

Figure 2: D.B. investigation, formal analysis,methodology and validation, D.B. and T.P. conceptualization, T.P. resources, L.D. formal analysis, methodology and validation

Figure 3: D.B. and K.T.D investigation, formal analysis, D.B. and T.P. conceptualization, T.P. resources, D.B. software, methodology and validation

Figure 4: L.D. and S.P. formal analysis,software, methodology and validation, D.B., L.D. and T.P. conceptualization, T.P. resources

Figure 5: D.B. investigation, formal analysis, software, methodology and validation, D.B. and T.P. conceptualization, Y.E.H resources.

Figure 6: D.B., L.D., L.S.. investigation, formal analysis, D.B. and L.S. software, methodology and validation, T.P. conceptualization, T.P. resources

Figure 7: D.B. and T.P. conceptualization, D.B. software and figure using BioRender

Supplementary figures 1-4: D.B. investigation, formal analysis, software, methodology and validation, D.B. and T.P. conceptualization

Supplementary figure 5: L.D., D.B. and A.M. formal analysis, software, methodology and validation, D.B. and T.P. conceptualization

Supplementary figure 6: D.B. investigation, formal analysis, software, methodology and validation, D.B. and T.P. conceptualization, Y.E.H resources.

Signed for and on behalf of the Author(s):	Print Name:	Date:
TP	Dr. Thomas Pulinilkunnil	June 25, 2021
