# Supplementary material for: Inhibiting BCKDK in triple negative breast cancer suppresses protein translation, impairs mitochondrial function, and potentiates doxorubicin cytotoxicity
Source: Cell Death Discov. 2021 Sep 15;7:241. doi: 10.1038/s41420-021-00602-0 (PMC8443725; doi:10.1038/s41420-021-00602-0)
Supplement: Supplementary file 9 — Authorship Addition Justification Approval Form [file 41420_2021_602_MOESM9_ESM.pdf]

SPRINGER NATURE

Change of authorship request form - Journals (pre-acceptance)

Important information. Please read.

- This form should be used by authors to request any change in authorship (adding/deleting authors) including changes in corresponding authors. This form should not be used for name changes. Please fully complete all sections. Use black ink and block capitals and provide each author's full name with the given name first followed by the family name.

- By signing this declaration, all authors guarantee that the order of the authors are in accordance with their scientific contribution, if applicable as different conventions apply per discipline, and that only authors have been added who made a meaningful contribution to the work.

- Please note, in author collaborations where there is formal agreement for representing the collaboration, it is sufficient for the representative or legal guarantor (usually the corresponding author) to complete and sign the Authorship Change Form on behalf of all authors, **next to the added/removed author(s). (Complete Section 3, followed by Section 6.)**
In author collaborations where there is no formal agreement for representing the collaboration and **there are more than 10 authors**, one may sign for all, provided the signer appends correspondence that attests that each of the authors have agreed to the change **and the added/removed authors sign the form**. **(Complete Section 3, followed by Section 6.)**

- Please note, we cannot investigate or mediate any authorship disputes. If you are unable to obtain agreement from all authors (including those who you wish to be removed) you must refer the matter to your institution(s) for investigation. Please inform us if you need to do this.

- If you are not able to return a fully completed form within **30 days** of the date that it was sent to the author requesting the change, we may have to withdraw your manuscript. We cannot publish manuscripts where authorship has not been agreed by all authors (including those who have been removed).

- Incomplete forms will be rejected.

- Please return/upload this form, fully completed, to the Journals Editorial Office. The Journal and/or Publisher will consider the information you have provided to decide whether to approve the proposed change in authorship. We may decide to contact your institution for more information or undertake a further investigation, if appropriate, before making a final decision.

SPRINGER NATURE

Change of authorship request form - Journals (pre-acceptance)

Section 1: Please provide the current title of manuscript

Manuscript ID no.: CDDISCOVERY-21-1626R

Title: Inhibiting BCKDK in triple negative breast cancer suppress protein translation, impair mitochondrial function, and potentiate doxorubicin cytot

Section 2: Please provide the previous authorship, in the order shown on the manuscript before the changes were introduced. Please indicate the corresponding author by adding (CA) behind the name.

	First name(s)	Family name	ORCID or SCOPUS id, if available
1st author	Dipsikha	Biswas	0000-0003-0990-9678
2nd author	Logan	Slade	
3rd author	Luke	Duffley	
4th author	Neil	Mueller	
5th author	Khoi	Thien Dao	
6th author	Angella	Mercer	
7th author	Yassine	El Hiani	
8th author	Petra	Kienesberger	0000-0001-9643-8338
9th author	Thomas	Pulinilkunnil (CA)	0000-0003-1228-893X
10th author			

Please use an additional sheet if there are more than 10 authors.

SPRINGER NATURE Springer Nature is one of the world's leading global research, educational and professional publishers, created in May 2015 through the combination of Nature Publishing Group, Palgrave Macmillan, Macmillan Education and Springer Science+Business Media.

SPRINGER NATURE

Change of authorship request form - Journals (pre-acceptance)

Section 3: Please provide a justification for change. Please use this section to explain your reasons for changing the authorship of your manuscript, e.g. what necessitated the change in authorship? Please refer to the (journal) policy pages for more information about authorship. Please explain why omitted authors were not originally included and/or why authors were removed on the submitted manuscript.

A new author Dr. Shanmugasundaram Pakkiriswami has been added to the manuscript for the following reasons;
Due to the departure of the first author to another position my postdoctoral fellow Dr. Pakkiriswami undertook the tasks of completing the revision for this manuscript along with my graduate student Mr. Logan Slade. Due to ongoing pandemic restrictions Dr. Pakkiriswami's entry to my laboratory was only possible after December 2020 and he overlapped with Dr. Biswas (first author) after the first submission of this manuscript to CDD. Dr. Pakkiriswami' s contributions is highlighted in the authorship contribution section. He supported cell culture, transcriptome data analysis and critically reviewing the revised submission.

Section 4: Proposed new authorship. Please provide your new authorship list in the order you would like it to appear on the manuscript. Please indicate the corresponding author by adding (CA) behind the name. <u>If the Corresponding Author has changed, please indicate the reason under section 3.</u>

	First name(s)	Family name (this name will appear in full on the final publication and will be searchable in various abstract and indexing databases)	Affiliated institute	E-mail address
1st author	Dipsikha	Biswas	Dalhousie University	dp369152@dal.ca
2nd author	Logan	Slade	Dalhousie University	Logan.Slade@Dal.Ca
3rd author	Luke	Duffley	Dalhousie University	lduffley@mun.ca
4th author	Neil	Mueller	Dalhousie University	nl444712@dal.ca
5th author	Khoi	Thien Dao	Dalhousie University	kh450940@dal.ca
6th author	Angella	Mercer	Dalhousie University	an219892@dal.ca
7th author	Shanmugasundaram	Pakkiriswami	Dalhousie University	sn374151@dal.ca
8th author	Yassine	El Hiani	Dalhousie University	yassine.elhiani@Dal.Ca
9th author	Petra	Kienesberger	Dalhousie University	pkienesb@dal.ca
10th author	Thomas	Pulinilkunnil (CA)	Dalhousie University	tpulinil@dal.ca

Please use an additional sheet if there are more than 10 authors.

SPRINGER NATURE Springer Nature is one of the world's leading global research, educational and professional publishers, created in May 2015 through the combination of Nature Publishing Group, Palgrave Macmillan, Macmillan Education and Springer Science+Business Media.

SPRINGER NATURE

Change of authorship request form - Journals (pre-acceptance)

Section 5: Author contribution, Acknowledgement and Disclosures. Please use this section to provide a new disclosure statement and, if appropriate, acknowledge any contributors who have been removed as authors and ensure you state what contribution any new authors made (if applicable per the journal or book (series) policy). Please ensure these are updated in your manuscript - after approval of the change(s) - as our production department will not transfer the information in this form to your manuscript.

New acknowledgements:

None

New Disclosures (financial and non-financial interests, funding):

None

New Author Contributions statement (if applicable per the journal policy):

Updated author contribution statement was attached in the revised submission. Dr. Pakkiriswami conducted formal analysis,software, methodology and validation for the transcriptome data in the revised submission. He also assisted in cell culture work to generate samples for UPLC MS spec analysis and proof reading the manuscript prior to formal submission.

State 'Not applicable' if there are no new authors.

SPRINGER NATURE Springer Nature is one of the world's leading global research, educational and professional publishers, created in May 2015 through the combination of Nature Publishing Group, Palgrave Macmillan, Macmillan Education and Springer Science+Business Media.

SPRINGER NATURE

Change of authorship request form - Journals (pre-acceptance)

Section 6: Declaration of agreement. *All* authors, unchanged, new and removed *must* sign this declaration.
(NB: Please print the form, (docu)-sign and return/upload a scanned copy. Please note that signatures that have been inserted as an image file are acceptable as long as it is handwritten. Typed names in the signature box are unacceptable.) *** Please delete as appropriate. Delete all of the bold if you were on the original authorship list and are remaining as an author.**

	First name	Family name		Signature	Date
1st author	Dipsikha	Biswas	I agree to the proposed new authorship shown in section 4 **/and the addition/removal*of my name to the authorship list** /and the proposed change in corresponding author	Dipsikha Biswas	July 09, 2021
2nd author	Logan	Slade	I agree to the proposed new authorship shown in section 4 **/and the addition/removal*of my name to the authorship list** /and the proposed change in corresponding author	Logan Slade	July 09, 2021
3rd author	Luke	Duffley	I agree to the proposed new authorship shown in section 4 **/and the addition/removal*of my name to the authorship list** /and the proposed change in corresponding author	Luke Duffley	July 09, 2021
4th authors	Neil	Mueller	I agree to the proposed new authorship shown in section 4 **/and the addition/removal*of my name to the authorship list** /and the proposed change in corresponding author	Neil Mueller	July 09, 2021
5th author	Khoi	Thien Dao	I agree to the proposed new authorship shown in section 4 **/and the addition/removal*of my name to the authorship list** /and the proposed change in corresponding author	[illegible]	July 09, 2021
6th author	Angella	Mercer	I agree to the proposed new authorship shown in section 4 **/and the addition/removal*of my name to the authorship list** /and the proposed change in corresponding author	Angella Mercer	July 09, 2021
7th author	Shanmugasundaram	Pakkiriswami	I agree to the proposed new authorship shown in section 4 **/and the addition/removal*of my name to the authorship list** /and the proposed change in corresponding author	[illegible]	July 09, 2021

SPRINGER NATURE Springer Nature is one of the world's leading global research, educational and professional publishers, created in May 2015 through the combination of Nature Publishing Group, Palgrave Macmillan, Macmillan Education and Springer Science+Business Media.

Change of authorship request form - Journals (pre-acceptance)

	First name	Family name		Signature	Date
8th author	Yassine	El Hiani	I agree to the proposed new authorship shown in section 4 /**and the addition/removal*of my name to the authorship list** /and the proposed change in corresponding author		July 09, 2021
9th author	Petra	Kienesberger	I agree to the proposed new authorship shown in section 4 /**and the addition/removal*of my name to the authorship list** /and the proposed change in corresponding author		July 09, 2021
10th author	Thomas	Pulinilkunnil	I agree to the proposed new authorship shown in section 4 /**and the addition/removal*of my name to the authorship list** /and the proposed change in corresponding author		July 09, 2021

Please use an additional sheet if there are more than 10 authors.

In case of author collaborations with formal agreement:

	Name of consortium/consortia	First name	Family name		Signature	Date
Representative/legal guarantor				I agree to the proposed new authorship shown in section 4 /**and the addition/removal*of my name to the authorship list** /and the proposed change in corresponding author		

Both added/removed authors should complete the information in the first table under Section 6.

---- End of form ----

SPRINGER NATURE Springer Nature is one of the world's leading global research, educational and professional publishers, created in May 2015 through the combination of Nature Publishing Group, Palgrave Macmillan, Macmillan Education and Springer Science+Business Media.
